# Therapeutics for Nipah virus disease: a systematic review to support prioritisation of drug candidates for clinical trials

**DOI:** 10.1016/j.lanmic.2024.101002

**Published:** 2025-05

**Authors:** Xin Hui S Chan, Ilsa L Haeusler, Bennett J K Choy, Md Zakiul Hassan, Junko Takata, Tara P Hurst, Luke M Jones, Shanghavie Loganathan, Elinor Harriss, Jake Dunning, Joel Tarning, Miles W Carroll, Peter W Horby, Piero L Olliaro

**Affiliations:** a Pandemic Sciences Institute Nuffield Department of Medicine, University of Oxford, Oxford, UK; b Centre for Human Genetics Nuffield Department of Medicine, University of Oxford, Oxford, UK; c Centre for Tropical Medicine and Global Health Nuffield Department of Medicine, University of Oxford, Oxford, UK; d International Severe Acute Respiratory and Emerging Infection Consortium University of Oxford, Oxford, UK; e Bodleian Health Care Libraries University of Oxford, Oxford, UK; f Department of Clinical Infection, Oxford University Hospitals NHS Foundation Trust, Oxford, UK; g Programme for Emerging Infections, Infectious Diseases Division, International Centre for Diarrheal Disease Research, Dhaka, Bangladesh; h Academic Foundation Programme, Kent Surrey and Sussex Deanery, London, UK; i Department of Infectious Diseases, Royal Free London NHS Foundation Trust, London, UK; j Mahidol-Oxford Tropical Medicine Research Unit, Faculty of Tropical Medicine, Mahidol University, Bangkok, Thailand

## Abstract

Nipah virus disease is a bat-borne zoonosis with person-to-person transmission, a case-fatality rate of 38–75%, and well recognised potential to cause a pandemic. The first reported outbreak of Nipah virus disease occurred in Malaysia and Singapore in 1998, which has since been followed by multiple outbreaks in Bangladesh and India. To date, no therapeutics or vaccines have been approved to treat Nipah virus disease, and only few such candidates are in development. In this Review, we aim to assess the safety and efficacy of the therapeutic options (monoclonal antibodies and small molecules) for Nipah virus disease and other henipaviral diseases to support prioritisation of drug candidates for further evaluation in clinical trials. At present, sufficient evidence exists to suggest trialling 1F5, m102.4, and remdesivir (alone or in combination) for prophylaxis and early treatment of Nipah virus disease. In addition to well designed clinical efficacy trials, in-vivo pharmacokinetic–pharmacodynamic studies are needed to optimise the selection and dosing of therapeutic candidates in animal challenge and natural human infection.

## Introduction

Nipah virus disease is a zoonotic infection acquired through contact with or ingestion of contaminated body fluids of infected mammals.^1^, ^2^, ^3^ Pteropid fruit bats (flying foxes) are the primary reservoir of Nipah virus. Secondary hosts include domestic animals^4^^,^^5^ (such as pigs, horses, and cows) and humans. Nipah virus also spreads through person-to-person transmission. Nipah virus disease occurs in all age groups.^1^^,^^6^ The clinical presentation of Nipah virus infection ranges from being asymptomatic^7^ to an acute respiratory syndrome and fatal encephalitis.^1^^,^^6^ After an incubation period of 4–14 days, fever, headache, and myalgia can occur, followed by shortness of breath and cough or confusion and seizures, which can rapidly progress to coma within 24–48 h.^1^^,^^6^ The case-fatality rate is estimated to be between 38% and 75%, and debilitating long-term neurological complications, such as paralysis, are common in individuals who have had Nipah virus infection.^1^^,^^8^

Nipah virus is part of the genus *Henipavirus*, along with Hendra virus, which also causes fatal encephalitis and respiratory diseases in horses and humans. Both Nipah and Hendra viruses are biosafety level 4 (BSL-4) pathogens that require the highest level of laboratory containment precautions. The other bat-borne members of the genus (Cedar and Kumasi viruses) are not known to cause human diseases.^9^

First identified in 1998, following an outbreak among pig farmers and abattoir workers in Malaysia^10^ and Singapore,^11^ Nipah virus is named after the Malaysian village from which the virus was first isolated. 283 cases of encephalitis and 109 deaths were recorded in that outbreak, with a case-fatality rate of 38·3%.^12^ The outbreak was contained with mass culling of over 1 million pigs and comprehensive modernisation of pig farming practice, including spatial separation of fruit tree plantations from pig farms.^13^

No further Nipah cases have been reported in Malaysia and Singapore since, and only one further outbreak of the Nipah virus Malaysia (NiV-M) strain has been reported in the Philippines in 2014,^14^ which was related to horse slaughter and consumption.

However, outbreaks of the Nipah virus Bangladesh (NiV-B) strain have been reported in Bangladesh^3^ and India (in the states of West Bengal^15^ and Kerala^16^) since 2001, with health-care workers^15^ and family members^17^ caring for infected patients emerging as another important risk group. Only NiV-B has known person-to-person transmission.

The highest mortality rates have been recorded in Bangladesh, where outbreaks occur almost every year in the winter following harvesting and consumption of contaminated raw date palm sap,^2^ a local delicacy. Since 2001, there have been 335 cases with 237 deaths in Bangladesh, with a case-fatality ratio of 70·7%.^3^ The 2023 outbreak in Bangladesh was the largest since 2015, with 14 cases and ten deaths. A second outbreak occurred 6 months later in Kerala, India, with six cases and two fatalities.^18^ Patient outcomes have not improved in the 25 years since the first reported outbreaks of the infection, reflecting the market failure typical of countermeasure development for a high-consequence pathogen.^19^

No approved vaccines or therapeutics are available for Nipah virus infection, and only a few candidates are in the development stage.^20^ In recognition of the urgent need for vaccines and therapeutics, Nipah virus infection has been a priority disease in the WHO R&D Blueprint since 2018.^21^ Clinical evaluation of vaccines and therapeutics for Nipah virus disease is restricted by the infeasibility of a controlled human infection model and small number of patients in sporadic outbreaks. Assessment of the effectiveness of therapeutics and vaccines is therefore reliant on animal challenge studies conducted in BSL-4 laboratory facilities.

This Review assessed the safety and efficacy of therapeutic options (monoclonal antibodies and small molecules) for Nipah virus and other Henipaviridae that cause human disease to support drug candidate prioritisation for evaluation in clinical trials.

## Methods

This systematic review was registered prospectively on the PROSPERO database (CRD42022346563) and adheres to the PRISMA 2020 reporting guidelines (appendix pp 28–30).

### Search strategy and selection criteria

An electronic literature search of the following bibliographic databases was conducted for journal articles, conference abstracts, and patents: PubMed, Ovid Embase, Ovid CAB Abstracts, Ovid Global Health, Scopus, Web of Science (all databases), and the WHO Global Index Medicus. “Henipavirus” or “Nipah” or “Hendra” along with “therapeutics” or “monoclonal” were the search terms for titles, abstracts, and subject headings, with synonyms and variant spellings serving as additional search terms.

The following trial registries were searched for clinical trials of henipavirus, Nipah virus, and Hendra virus at all stages of recruitment: Cochrane Central Register of Controlled Trials, ClinicalTrials.gov, and the WHO International Clinical Trials Registry Platform. The Trip database and WHO website were searched for guidelines and reports. The full search strategies are detailed in the appendix (pp 3–4).

All searches were updated on Sept 5, 2024, and had no language or publication date criteria. The references identified were imported into EndNote, deduplicated, and then screened against eligibility criteria. Reference lists of the eligible records were checked for additional relevant studies.

### Eligibility criteria

Studies containing primary data on the safety or efficacy, or both, of monoclonal antibodies (in vivo) or small molecules (in vivo or in vitro) for the treatment or prophylaxis, or both, of Nipah or Hendra infections, or both, were included. Studies on candidates without therapeutic applications (eg, monoclonal antibodies for diagnostics) or with only in-silico data were excluded.

### Data extraction

We extracted data on the viruses studied, study characteristics (funder, year, location, and design), intervention characteristics (drug, dose, route, and administration timepoints), efficacy outcomes (all measures and all timepoints), and safety outcomes (all measures and all timepoints). Study investigators and experts were contacted for further information, as needed.

### Data analysis

The review pilot identified substantial heterogeneity in study designs, outcome measures, and reporting. Quantitative data synthesis was not deemed possible. All available data were, therefore, prespecified to be summarised in a tabular format by individual therapeutic candidates as a narrative synthesis prioritising clinical and animal studies.

### Quality assessment

Risk of Bias assessment was undertaken for the study designs using standardised tools: Risk of Bias 2 (RoB 2^22^) for randomised clinical trials (RCTs); Risk of Bias in non-randomised studies of interventions (ROBINS-I^23^) for non-randomised clinical studies; and systematic review centre for laboratory animal experimentation (SYRCLE^24^) for animal studies.

### Review team and tools

At least two independent reviewers (XHSC, ILH, BJKC, MZH, JTak, TPH, LMJ, and SL) performed screening (titles and abstracts, followed by full texts), discussed study eligibility, extracted data, and undertook Risk of Bias assessment using Covidence.

## Results

### Studies included

We identified 59 eligible studies (figure): 14 on monoclonal antibodies with clinical or animal data, or both (table 1 and appendix pp 7–11); 26 on small molecules with clinical or animal data, or both (table 2 and appendix pp 12–20); and 19 on small molecules with in-vitro data only (appendix pp 21–23).

**Figure:**
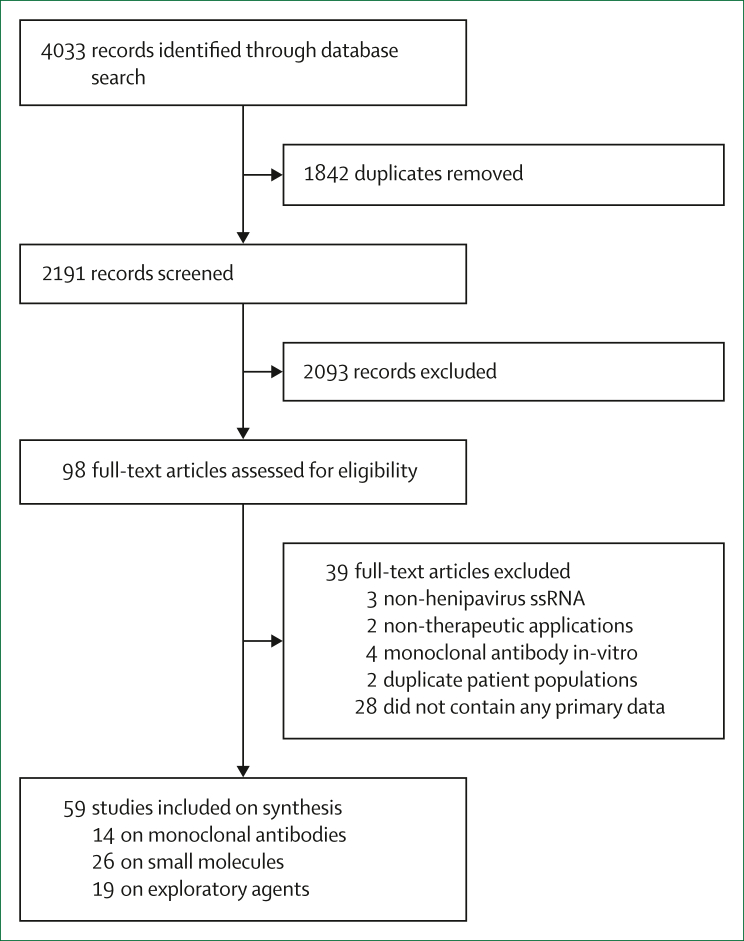
PRISMA flow chart

**Table 1:** Therapeutic monoclonal antibodies against NiV and HeV by drug name (and mechanism)

	Developer	Reference	Study design	Funder
m102.4 (anti-HeV-G)	Uniformed Services University, USA	Sahay et al (2020)^25^	Clinical: compassionate use in postexposure prophylaxis during NiV outbreak in Kerala, India (n=1)	US National Institutes of Health
		Playford et al (2020)^26^	Clinical: healthy adult volunteers (18–50 years) phase 1 dose-escalation randomised controlled trial for safety, tolerability, and pharmacokinetics in Brisbane, Australia (n=40)	
		Mire et al (2016)^27^	Animal: African green monkey challenge with NiV-B for efficacy and safety (n=11) 2.5 × 10^5^ PFU intratracheal plus 2·5 × 10^5^ PFU intranasal	
		Geisbert et al (2014)^28^	Animal: African green monkey challenge with NiV-M for efficacy and safety (n=16) 5 × 10^5^ PFU intratracheal	
		Bossart et al (2011)^29^	Animal: African green monkey challenge with HeV for efficacy and safety (n=14) 4 × 10^5^ TCID_50_ intratracheal Animal: African green monkey pharmacokinetics (n=4)	
		Bossart et al (2009)^30^	Animal: ferret challenge with NiV-M for efficacy (n=8) 5 × 10^3^ TCID_50_ oronasal	
		Zhu et al (2008)^31^	Animal: ferret pharmacokinetics (n=4)	
1F5 and 12B2 (anti-NiV-F)		Zeitlin et al (2024)^32^	Animal: African green monkey challenge with NiV-B for efficacy (n=13) 4 × 10^4^ PFU intranasal	
			Animal: hamster challenge with NiV-B for efficacy (n=16) 5 × 10^6^ PFU intranasal	
h5B3.1 (anti-NiV-F)		Mire et al (2020)^33^	Animal: ferret challenge with NiV-M or HeV for efficacy (n=11) 5 × 10^3^ PFU intranasal	
NiV41 and NiV41-6 (anti-NiV-RBP)	Wuhan Institute of Virology, China	Chen et al (2024)^34^	Animal: hamster challenge with NiV-B for efficacy (n=12) 10^5^ TCID_50_ intraperitoneal	Chinese Academy of Sciences
			Animal: hamster challenge with NiV-M for efficacy (n=48) 1000 LD_50_ intraperitoneal	
HENV-103, HENV-117, HENV-58, HENV-98, and HENV-100 (anti-HeV-RBP)	Vanderbilt University, USA	Doyle et al (2021)^35^	Animal: hamster challenge with NiV-B for efficacy (n=46) 5 × 10^6^ PFU intranasal	US National Institutes of Health
HENV-26 and HENV-32 (anti-HeV-RBP)		Dong et al (2020)^36^	Animal: ferret challenge with NiV-B for efficacy (n=13) 5 × 10^3^ PFU intranasal	
NipGIP1.7 and Nip3B10 (anti-NiV-G) NipGIP35 and NipGIP3 (anti-NiV-F)	INSERM, France	Guillaume et al (2006)^37^	Animal: hamster challenge with NiV-M for efficacy, dose titration, and therapeutic time window (n=124) 7·5 × 10^2^ PFU (100 LD_50_) intraperitoneal	Aventis Pharma, Bayer Pharma, INSERM, and Institut Pasteur
NipGIP35, NipGIP3, NipGIP21, and NipGIP7 (anti-NiV-F)		Guillaume et al (2009)^38^	Animal: hamster challenge with HeV for efficacy and dose titration (n=54) 10^3^ PFU (100 LD_50_) intraperitoneal	

HeV=Hendra virus. INSERM=Institut National de la Santé et de la Recherche Médicale. LD_50_=median lethal dose. NiV-B=Nipah virus Bangladesh. NiV-M=Nipah virus Malaysia. PFU=plaque-forming units. RBP=receptor binding protein. TCID_50_=median tissue culture infectious dose.

Please see appendix pp 7–11 for the full table.

**Table 2:** Therapeutic small molecules against NiV and HeV by drug name (and mechanism)

	Reference	Study design	Funder
Ribavirin (nucleoside analogue prodrug)	Warrier et al (2020)^39^	Clinical: compassionate use for treatment in NiV outbreak in Kochi, India, 2019 (n=1)	NA
	Chandni et al (2020)^40^	Clinical: compassionate use for treatment in NiV outbreak in Kerala, India, 2018 (n=12: 6 treated, 6 untreated)	
	Banerjee et al (2019)^41^	Clinical: compassionate use for postexposure prophylaxis of health-care workers during NiV outbreak in Kerala, India, 2018 (n=8)	
	Kumar et al (2019)^42^	Clinical: compassionate use for treatment in NiV outbreak in Kerala, India, 2018 (n=5)	
	Playford et al (2010)^43^	Clinical: compassionate use during HeV outbreak in Australia, 2008, for treatment (n=2) and postexposure prophylaxis (n=1)	
	Chong et al (2001)^44^	Clinical: compassionate use for treatment in NiV outbreak in Malaysia, 1998–99 (n=194: 140 treated, 54 untreated)	
	Rockx et al (2010)^45^	Animal: African green monkey challenge with HeV (n=12) 4 × 10^5^ TCID_50_ intratracheal for efficacy	
Ribavirin (nucleoside analogue prodrug), 6-azauridine (orotidine monophosphate decarboxylase inhibitor), and rintatolimod (toll-like receptor 3 agonist interferon inducer)	Georges-Courbot et al (2006)^46^	Animal: hamster challenge with NiV-M for efficacy (n=18) 350 × LD_50_ intraperitoneal Animal: hamster challenge with NiV-M for efficacy (n=18) 35 × LD_50_ intraperitoneal	NA
Ribavirin (nucleoside analogue prodrug) and chloroquine (lysosome alkalinisation)	Freiberg et al (2010)^47^	Animal: hamster challenge with NiV-M (n=41) and HeV (n=20) for efficacy (n=85) 10^4^ TCID_50_ intraperitoneal	US NIH
Chloroquine (lysosome alkalinisation)	Pallister et al (2009)^48^	Animal: ferret challenge with NiV-M for efficacy and pharmacokinetics (n=8) 5 × 10^3^ TCID_50_ oronasal	US NIH
Remdesivir (nucleoside analogue)	de Wit et al (2023)^49^	Animal: African green monkey challenge with NiV-B for efficacy (n=18) 10^5^ TCID_50_ intranasal plus 10^5^ TCID_50_ intratracheal	US NIH
	Lo et al (2019)^50^	Animal: African green monkey challenge with NiV-B for efficacy (n=8) 10^5^ TCID_50_ intranasal plus 10^5^ TCID_50_ intratracheal	
	Jordan et al (2017)^51^	Animal: African green monkey challenge with NiV-B for efficacy Lethal dose (unspecified)	
Favipiravir (nucleoside analogue prodrug)	Dawes et al (2018)^52^	Animal: hamster challenge with NiV-M for efficacy (n=18) 10^4^ PFU intraperitoneal	US NIH
Griffithsin (fusion and cell entry inhibitor)	Lo et al (2020)^53^	Animals: hamster challenge with NiV-B for efficacy (n=65) 10^7^ TCID_50_ intranasal	US NIH and US CDC
Periodate heparin (competitive inhibitor of transinfection)	Mathieu et al (2015)^54^	Animal: hamster challenge with NiV-M for efficacy (n=15) 500 × LD_50_ intraperitoneal	INSERM
Fusion inhibitory lipopeptides (fusion and cell entry inhibitors): VIKI-dPEG4-Chol, VIKI-dPEG4-Toco, VG-PEG24-chol, and VIKI-PEG4-chol	Mathieu et al (2018)^55^	Animal: hamster challenge with NiV-M for efficacy (n=38) 10^6^ PFU (100 × LD_50_) intranasal Animal: African green monkey challenge with NiV-M for efficacy (n=10) 2 × 10^7^ PFU intratracheal Animal: African green monkey biodistribution (n=4)	US NIH and INSERM
	Mathieu et al (2017)^56^	Animal: hamster challenge with NiV-M for efficacy (n=13) 100 × LD_50_ intraperitoneal Animal: Hamster biodistribution (n=6)	
	Porotto et al (2010)^57^	Animal: hamster challenge with NiV (strain unspecified) for efficacy (n=35) 100 × LD_50_ intraperitoneal	
Defective interfering particles (virus-like particles containing defective interfering genomes that inhibit replication): DI-07, DI-10, DI-14, and DI-35	Welch et al (2022)^58^	Animal: hamster challenge with NiV-M for efficacy (n=153) Experiment 1: 10^4^ TCID_50_ intraperitoneal Experiment 2: 10^6^ TCID_50_ intranasal	US CDC
Ceftriaxone (bacterial cell wall synthesis inhibitor), clarithromycin (bacterial protein synthesis inhibitor), and aciclovir (nucleoside analogue)	Paton et al (1999)^11^	Clinical: empirical syndromic treatment during outbreak in Singapore, 1999 (n=11)	NA

CDC=Centers for Disease Control and Prevention. dPEG=discrete polyethylene glycol. HeV=Hendra virus. INSERM=Institut National de la Santé et de la Recherche Médicale. LD_50_=median lethal dose. NA=not available. NIH=National Institutes of Health. NiV-B=Nipah virus Bangladesh. NiV-M=Nipah virus Malaysia. PFU=plaque-forming units. TCID_50_=median tissue culture infectious dose.

Please see appendix pp 12–16 for the full table.

### Monoclonal antibodies

The 14 articles on monoclonal antibodies reported data on eight sets of antibodies from four research groups. Seven of the articles were on m102.4, an anti-Hendra virus (HeV)-G glycoprotein antibody (table 1 and appendix pp 7–11). m102.4 was the only Nipah drug candidate with clinical data from an RCT^26^ (phase 1) and in-vivo data from more than one animal species (African green monkeys^27^, ^28^, ^29^ and ferrets^30^^,^^31^) challenged with different henipaviruses (NiV-B,^27^ NiV-M,^28^^,^^30^ and HeV^29^; table 1).

The available data in humans support the safety of m102.4. A first-in-human dose-escalation randomised placebo-controlled trial^26^ of intravenous m102.4 (single doses of 1−20 mg/kg along with two doses of 20 mg/kg 72 h apart) in 40 healthy adult volunteers followed up for approximately 4 months did not report any serious adverse events. The frequency of adverse events, of which headache was the most common, was similar between the treatment and placebo groups. No anti-m102.4 antibodies were detected.

Before the trial, 14 individuals aged 8–59 years received m102.4 as postexposure prophylaxis on compassionate grounds for Hendra virus infection (n=13) in Australia and for Nipah virus infection (n=1) in the USA.^26^ Among the individuals, two had infusion-related febrile reactions that were attributed to an early production process of the antibody.^26^ In addition, one outbreak report described a single patient receiving m102.4 as postexposure prophylaxis in Kerala, India, in 2018.^25^ The patient was reported to have recovered completely, but no further details were provided.

Four animal challenge studies showed the efficacy of m102.4 in preventing death and severe disease in all treated animals when a single dose of m102.4 was administered to ferrets 10 h after oronasal NiV-M inoculation^30^ (n=3) or in a two-dose regimen administered 48 h apart in monkeys, starting within 5 days after intratracheal NiV-M^28^ (n=12) or HeV^29^ (n=12) challenge. In comparison, all control animals died within 8–10 days in the three studies. However, the treatment time window for the two-dose regimen after NiV-B challenge in monkeys^27^ was shorter than that after NiV-M challenge and HeV challenge, with only the animals treated within 3 days (n=6) from inoculation surviving to the end of the study and those treated on days 5 and 7 (n=2) showing similar outcomes as the controls (n=2).

The developers of m102.4 also investigated anti-NiV-F glycoprotein monoclonal antibodies: h5B.3^33^ and 1F5.^32^ When administered intraperitoneally to ferrets in a regimen of two 20 mg/kg doses given 48 h apart starting within 3 days of intranasal challenge with NiV-M (n=6) or HeV (n=3), h5B.3 protected all treated ferrets from severe disease.^33^

1F5 was selected in favour of 12B2 (another anti-NiV-F monoclonal antibody) for its superior protection in NiV-B-infected hamsters.^32^ When administered intraperitoneally 24 h after intranasal inoculation, 1F5 prevented death in all five hamsters compared with the partial protection conferred after 12B2 treatment.^32^ 1F5 also provided complete protection to NiV-B-inoculated monkeys at 5 days after challenge when administered intravenously at doses of 25 mg/kg (n=6) and 10 mg/kg (n=3), whereas intravenous m102.4 at a dose of 25 mg/kg resulted in the survival of only one of the six monkeys till the study endpoint.^32^ The data indicated that 1F5 has a longer treatment time window (5 days) than m102.4 (3 days) for NiV-B in monkeys.^32^

Anti-NiV receptor binding protein antibody NiV41 and its mature form NiV41–6 have been studied in NiV-challenged hamsters.^34^ NiV41–6 provided complete protection to hamsters against death at a dose of 10 mg/kg (n=6) and only partial protection at 3 mg/kg (n=6) when administered intraperitoneally 24 h before intraperitoneal NiV-M inoculation. When NiV41–6 was administered 3 h, 3 h and 3 days, and 1 day and 3 days after challenge (n=6 each group), protection was partial and declined the later the monoclonal antibody was initiated after challenge.^34^

Two studies^35^^,^^36^ described two sets of anti-HeV receptor binding protein antibodies. HENV-26 (n=5) and HENV-32 (n=5) administered intraperitoneally as two doses of 15 mg/kg on days 3 and 5 after intranasal NiV-B challenge each protected ferrets from death and severe disease compared with that observed in the controls (n=3).^36^ HENV-103 and HENV-117 protected all hamsters from intranasal NiV-B challenge in combination (n=5), but not individually (n=5 each), nor as two bispecific antibodies of different designs (n=5 each).^35^

Two other studies^37^^,^^38^ described two groups of anti-NiV-F and anti-NiV-G protein antibodies and provided details on protection, dose titration, and therapeutic time window; one study involved 124 hamsters^37^ and the other involved 54 hamsters.^38^ However, no further studies on these specific anti-NiV-F and anti-NiV-G protein antibodies have been conducted in the subsequent two decades.

### Small molecules

In this Review, 26 articles on small molecules with in-vivo (table 2 and appendix pp 12–16) and in-vitro (appendix pp 17–20) data were identified. The articles described ten potential therapeutics and one group of syndrome-directed broad-spectrum empirical antimicrobials.

Ten of the 26 studies were on ribavirin, a repurposed nucleoside analogue prodrug, and included six clinical case series for treatment^39^^,^^40^^,^^42^, ^43^, ^44^ and postexposure prophylaxis^41^^,^^43^ for Nipah^39^, ^40^, ^41^, ^42^^,^^44^ and Hendra^43^ virus outbreaks (table 2); three animal challenge studies of African green monkeys (HeV^45^ only) and hamsters (HeV^47^ and NiV-M^46^^,^^47^; table 2); and three sets of in-vitro experiments with HeV^47^^,^^59^ and NiV-M^46^^,^^47^ (appendix pp 17–20).

The small number of patients administered ribavirin (n<10 in all, except the NiV-M outbreak in Malaysia^44^) and the pragmatic observational designs of the case series precluded definitive statements about clinical efficacy. Dose regimens were different among the four publications^40^^,^^41^^,^^43^^,^^44^ reporting on clinical efficacy and were not reported in the remaining two publications.^39^^,^^42^ All eight health-care workers in the only postexposure prophylaxis case series^41^ of ribavirin did not complete the prescribed course due to adverse effects: six of the eight had symptoms (such as fatigue or headache) or transient laboratory abnormalities (increased bilirubin or decreased haemoglobin concentrations, or both).

In the three animal studies, when administered intraperitoneally^46^^,^^47^ or subcutaneously^45^^,^^46^ at 24 h before challenge or within 12 h after challenge, ribavirin at 50–150 mg/kg per day delayed but did not prevent death or signs after NiV-M inoculation in hamsters^46^^,^^47^ (n=17) or HeV inoculation in African green monkeys^45^ (n=6), compared with that observed in the untreated controls. Ribavirin at 60 mg/kg per day neither delayed nor prevented death in HeV-challenged hamsters (n=5).^47^ Systemic toxicity owing to high-dose 200 mg/kg per day of intraperitoneal ribavirin was observed in infected and uninfected (control) hamsters, thus necessitating euthanasia.^47^

In-vitro experiments of ribavirin assessed viral replication through virus yield reduction,^59^ cytopathic effect,^46^ and dose–response^47^ assays in NiV-M-infected and HeV-infected Vero^46^^,^^59^ and HeLa^47^ cells. The ribavirin doses used to achieve 58-fold^59^ or 100% reductions^46^^,^^47^ in viral yield were high (50–409 μM) compared with the half-maximal inhibitory concentrations^47^ for NiV-M (4·18 μM) and HeV (4·96 μM).

Three studies assessed the widely used 4-aminoquinoline antimalarial chloroquine:^47^^,^^48^^,^^60^ two animal challenge studies^47^^,^^48^ (table 2) and two sets of in-vitro experiments^47^^,^^60^ (appendix pp 17–20). Ferrets administered 25 mg/kg per day chloroquine intravenously 24 h before (n=3) and 10 h after (n=3) NiV-M challenge presented disease courses identical to those observed in the controls.^48^ Hamsters inoculated with NiV-M and HeV and then treated intraperitoneally with 50 mg/kg chloroquine 6 h after challenge on alternate days as monotherapy (n=5 per virus) died earlier than the untreated controls, and hamsters treated in combination with ribavirin 30 mg/kg intraperitoneally twice a day (n=5 per virus) died at the same time as untreated controls.^47^ Chloroquine at 50 mg/kg per day intraperitoneally was also ineffective.^47^ Higher doses of 100 and 150 mg/kg per day of intraperitoneal chloroquine were consistently lethal by day 2 in both infected and uninfected hamsters.^47^

Four articles and one abstract on remdesivir, a nucleoside analogue, reported three NiV-B challenge studies in African green monkeys assessing intravenous administration^49^, ^50^, ^51^ (table 2) and in-vitro data from multiple assays on both the intravenous^61^ and oral^62^ formulations (appendix pp 17–20). Remdesivir 10 mg/kg per day administered from day 1 after challenge protected all four African green monkeys from death.^50^ Controls were all euthanised for disease severity after respiratory signs.^50^ The protective effect of remdesivir was time and dose dependent, with only partial protection when the treatment was started 3 days after challenge and declined further with dose reduction to 3 mg/kg.^49^ Reporter virus, cytopathic effect, and virus yield reduction assays for intravenous remdesivir (GS5734)^61^ and oral remdesivir (GS441524)^62^ and viral antigen reduction and minigenome assays for GS5734^61^ were performed in multiple cell types, including HeLa and human small airway epithelial cells. The mean half-maximal effective concentration (EC_50_) values were submicromolar for both and an order of magnitude lower for GS5734 (0·029–0·066 μM)^61^ than those for GS441524 (0·19–0·95 μM).^62^

The single study on favipiravir,^52^ a nucleoside analogue prodrug, contained data from an NiV-M hamster challenge study (table 2) and in-vitro assays (appendix pp 17–20). Hamsters administered 600 mg/kg subcutaneous favipiravir immediately after challenge, followed by a maintenance dose of 300 mg/kg favipiravirorally twice a day (n=5) or subcutaneously daily (n=5) for 13 days, survived without clinical signs or detectable pathology or viral antigen on necropsy, whereas all the controls died or were euthanised because of disease severity by day 5–6. The doses used in the virus yield (100 μM) assay to attain 100% viral reductions and in the delayed treatment (250 μM) assay to attain ten-fold (at 1 h after infection) viral reductions were high. The EC_50_ values were 11·7 μM for HeV, 14·8 μM for NiV-B, and 44·2 μM for NiV-M.^52^

Six other groups of small molecules were studied, none of which provided complete protection from death at the doses used in the animal challenge models. 6-Azauridine, the nucleoside analogue metabolite of the previously licensed azaribine, delayed the mean time to death by approximately 1 day but did not prevent death when administered immediately before a full-dose NiV-M challenge (350 × median lethal dose [LD_50_]) as a 175 mg/kg per day continuous subcutaneous infusion for 14 days in hamsters.^46^ Rintatolimod, a toll-like receptor 3 agonist, provided partial protection at 3 mg/kg per day intraperitoneal administration for 10 days at 2 h after a low-dose (35 × LD_50_) NiV-M inoculation^46^ (table 2 and appendix pp 12–16).

Periodate heparin, an experimental glycosaminoglycan competitive inhibitor of transinfection, protected one of the five hamsters challenged with NiV-M subcutaneously at a dose of 10 mg/kg per day for 12 days from the day of infection.^54^ Despite promising in-vitro results, experimental cell entry inhibitors such as the lectin griffithsin (oxidation-resistant and trimeric monomer)^53^ and fusion inhibitory lipopeptides (cholesterol-based and tocopherol-based)^55^^,^^57^ administered at a dose of 10 mg/kg per day intranasally (hamsters^55^, ^56^, ^57^) or intratracheally (African green monkeys^55^) prevented death in up to half of each group of the NiV-B-challenged^53^ or NiV-M-challenged^55^^,^^56^ animals (table 2). Defective interfering virus particles administered intraperitoneally or intranasally also showed partial efficacy in NiV-M-challenged hamsters,^58^ whereas the virus yield reduction assays presented an order of magnitude greater reduction in NiV-M-infected Vero cells than in NiV-B-infected ones^63^ (appendix pp 17–20).

### Risk of Bias

Most in-vivo studies had critical (six of eight case series) or high (18 of 26 animal studies) Risks of Bias. For animal studies, the high Risk of Bias was primarily because information related to most Risk of Bias domains had not been reported in study publications. Only three studies were assessed to have a low Risk of Bias: one RCT (of healthy volunteers),^26^ an outbreak report of a single case,^39^ and an NiV-B challenge study in African green monkeys.^50^ The remaining eight^25^^,^^30^^,^^32^, ^33^, ^34^^,^^49^^,^^54^^,^^64^ studies had an unclear Risk of Bias (appendix pp 26–27).

## Discussion

To the best of our knowledge, this Review is the most detailed study of the therapeutics landscape for Nipah and Hendra virus diseases with the specific aim of supporting drug candidate prioritisation for clinical trials. We did not identify any ongoing or completed therapeutic efficacy RCTs for Nipah or Hendra virus infection.

The pipeline of therapeutics that can be deployed rapidly at the outset of a henipavirus outbreak is restricted to a few monoclonal antibodies and repurposed small molecules with efficacy data from animal challenge models (table 3). The comparative advantages of monoclonal antibodies and small molecules are summarised in table 4.

**Table 3:** Clinical prioritisation of therapeutic candidates against NiV and HeV diseases

	Efficacy	Safety	Feasibility	Clinical prioritisation and proposed further evaluation
**Monoclonal antibodies (see also** table 1**)**				
m102.4 (anti-HeV-G)	Protected monkeys from death and pathology when administered as two doses 48 h apart, starting on day 1 or 3 after NiV-B^27^ (n=6) challenge or on day 1, 3, or 5 after NiV-M^28^ (n=12) and HeV^29^ (n=12) challenge. Also protected ferrets from death when administered 10 h after NiV-M^30^ (n=3) challenge	No serious adverse events, similar rate of mild treatment emergent adverse events between treatment and placebo groups, and no anti-m102.4 antibodies in phase 1 RCT in healthy adults (n=30 treated)^26^	High cost of goods,^20^ low drug supply, parenteral route only	High priority. Phase 2a postexposure prophylaxis or early treatment RCT, or both, during NiV or HeV disease outbreak. Shorter treatment window for NiV-B than for NiV-M. Dose optimisation for cost recommended.
1F5 (anti-NiV-F)	Protected monkeys from death and alleviated symptoms and viraemia when administered at doses of 25 mg/kg (n=6) and 10 mg/kg (n=3) on day 5 after NiV-B challenge, as compared with m102.4, which provided only partial protection at a dose of 25 mg/kg (1 of 6)^32^	No human studies to date. No safety data reported in animal studies	High cost of goods,^20^ low drug supply, parenteral route only	High priority. Phase 1 first-in-human RCT for safety and pharmacokinetics. Longer treatment window than that for m102.4 in animals with NiV-B. Dose optimisation for cost recommended.
h5B.3 (anti-NiV-F)	Protected ferrets from death but not minor clinical signs when administered at a dose of 20 mg/kg in two doses 48 h apart, starting on day 1 or 3 after NiV-M (n=6) or HeV (n=3) challenge^33^	No human studies to date. No safety data reported in animal studies.	High cost of goods,^20^ low drug supply, parenteral route only	Intermediate priority. Monkey studies with NiV-B challenge. Dose optimisation for cost necessary
NiV41-6 (anti-NiV-RBP)	Protected hamsters from death when administered at a dose of 10 mg/kg (n=6) 24 h before NiV-M challenge^34^			
HENV-26 (anti-HeV RBP)	Protected ferrets (n=5) from death, symptoms, and viraemia when administered at a dose of 15 mg/kg on days 3 and 5 after NiV-B challenge^36^			
HENV-103 plus HENV-117 (anti-HeV RBP)	HENV-103 plus HENV-117 cocktail (5 mg/kg each) protected hamsters (n=5) from death when administered a day after NiV-B challenge^35^			
**Small molecules (see also** table 2**)**				
Remdesivir (nucleoside analogue)	Protected monkeys (n=4) from death at a dose of 10 mg/kg when administered from day 1 after NiV-B challenge for 12 days,^50^ but provided only partial protection when administered from day 3 after challenge, with fewer monkeys protected at a dose of 3 mg/kg (2 of 6) than that of 10 mg/kg (4 of 6);^49^ in-vitro EC_50_ values=0·029–0·066 μM^61^	Sinus bradycardia and hepatotoxicity observed in humans^65^	Approved globally for COVID-19 Less affordable,^20^ parenteral route with oral formulation in development^62^	High priority. Phase 2a postexposure prophylaxis or early treatment RCT, or both, during NiV or HeV outbreak. Short treatment window for NiV-B. Dose optimisation for efficacy recommended.
Favipiravir (nucleoside analogue)	Fully protected hamsters (n=10) from death and alleviated symptoms and pathology when administered daily from time of NiV-M challenge; in-vitro EC_50_ values=11–44 μM^52^	Lethal toxicity in dogs and monkeys (>1 g/kg),^66^ teratogenicity across four animal species,^66^ transient hyperuricaemia in humans^66^^,^^67^	Approved in Japan for novel influenza.^66^ Affordable,^20^ oral route, but non-linear pharmacokinetics complicates dosing.^68^	Intermediate priority. Monkey study with NiV-B challenge. Dose optimisation for efficacy necessary.
Ribavirin (nucleoside analogue)	Delayed but did not prevent death when administered before or within 12 h to monkeys (n=6) after HeV^45^ challenge and to hamsters (n=17) after NiV-M^46^^,^^47^ challenge; in-vitro IC_50_ values=4·2–5·0 μM^47^	Dose-dependent toxicity in hamsters (>100 mg/kg)^47^ and humans^41^ restricts safety and tolerability	Approved globally for chronic hepatitis C. Affordable^20^ but equivocal risk-to-benefit ratio.	Intermediate priority. Monkey study with NiV-B challenge. Dose optimisation for safety and efficacy crucial. Time-to-event outcome measure when in phase 2a RCT.
Chloroquine (4-amino-quinoline)	Did not protect ferrets^48^ (n=6) and hamsters^47^ (n=19) from death when administered as monotherapy before or within 12 h after NiV-M or HeV challenge	Dose-dependent lethal toxicity in hamsters (>100 mg/kg)^47^ and humans (>3 μM plasma)^69^	Approved globally for malaria. Affordable^20^ but unfavourable risk-to-benefit ratio.	Low priority. Should not be used for the prophylaxis or treatment of NiV or HeV infection.

EC_50_=50% maximal effective concentration. HeV=Hendra virus. IC_50_=50% maximal inhibitory concentration. NiV-B=Nipah virus Bangladesh. NiV-M=Nipah virus Malaysia. RBP=receptor binding protein. RCT=randomised controlled trial.

High priority indicates that ample evidence exists for efficacy and safety for phase 2a trials. Intermediate priority indicates that further evidence is required for efficacy or potential major limitations in safety or feasibility, or both. Low priority indicates that no evidence exists for efficacy and major limitations in safety or feasibility, or both.

**Table 4:** Comparison of the advantages of monoclonal antibodies and small molecules

	Monoclonal antibodies	Small molecules
Efficacy	Highly specific	Less specific
Safety, including in pregnancy or lactation and other immunosuppression	Specificity confers low potential for off-target adverse effects Typically safe in pregnancy or lactation and immunosuppression	Higher potential for off-target adverse effects Often associated with hepatotoxicity, cardiotoxicity, or teratogenicity
Duration of effect	Half-life-extending mutations can prolong protection from 1 month to 3–6 months	Elimination half-lives of leading nucleoside analogue candidates between 1 and 5 h
Route of administration	Parenteral	Parenteral or oral, or both
Pharmacokinetic profile	Typically linear	Can be linear or non-linear
Cost	Higher (US$1000–2000)	Lower (US$10–100)
Development timeline	Shorter	Longer

Of the monoclonal antibodies, only m102.4 has been studied in humans, with safety and pharmacokinetic data from a phase 1 RCT in healthy adults.^26^ 1F5 is also progressing to phase 1 safety testing. The longer therapeutic time window within which complete protection is seen with 1F5 (5 days) in NiV-B-infected monkeys than that with m102.4 (3 days) would be of substantial clinical interest if validated in human trials in India and Bangladesh. Further pharmacokinetic studies of monoclonal antibody candidates to identify minimal doses for efficacy could help to make scale-up more cost-effective.

Of the small molecules, animal efficacy data were supportive for remdesivir and favipiravir, equivocal for ribavirin, and not supportive for chloroquine. Remdesivir was the only small molecule with in-vivo data from challenge with NiV-B,^49^^,^^50^ a strain closely related to those causing the Nipah outbreaks in Bangladesh and India.^70^ In addition, remdesivir has accumulated acceptable safety data from its widespread intravenous use against COVID-19.^71^ The therapeutic time window of remdesivir seen in NiV-B-infected monkeys is short,^49^ with protection declining from 3 days after inoculation;^49^ moreover, the therapeutic time window of remdesivir requires confirmation in humans.

Although favipiravir prevented death in NiV-M-challenged hamsters after a subcutaneous loading dose followed by subcutaneous or oral maintenance doses^52^ and could be an attractive choice for prophylaxis after exposure with a licensed oral formulation,^66^ the non-linear clinical pharmacokinetics of favipiravir seen in Ebola virus disease,^72^ influenza,^73^ and COVID-19^68^ necessitate further dose optimisation before inclusion in trials on Nipah virus disease. The non-linearity is thought to be explained by the concentration-dependent aldehyde oxidase inhibition reproducible in non-human primates,^74^ and any additional infection-specific contribution remains unclear as of now. Pharmacokinetic studies of parenteral (including intravenous^75^) administration in NiV-B-inoculated non-human primates would be the next key step in favipiravir evaluation. Notably, favipiravir is associated with teratogenicity in four animal species,^66^ and further data on the safety of favipiravir in humans are needed.^67^^,^^76^

Ribavirin prolongs survival but does not prevent death in monkeys and hamsters challenged with HeV^45^ and NiV-M;^46^^,^^47^ in fact, ribavirin is toxic to hamsters at high doses.^47^ Clinical tolerability issues (fatigue, anaemia, and hyperbilirubinaemia)^41^ are further likely to reduce adherence to a postexposure prophylaxis regimen containing ribavirin. Clinical reports of ribavirin in henipavirus outbreaks have all been observational, with dosing based on that used for Lassa fever.^44^ Clinical and pharmacokinetic meta-analyses of studies on ribavirin treatment for Lassa fever highlight the absence of robust data for its effectiveness,^77^ and conventional dosing regimens are unlikely to reliably achieve the serum concentrations required to inhibit Lassa virus replication.^78^

Pharmacokinetic modelling for the existing ribavirin dosing regimens for Nipah and Hendra virus diseases is ongoing. Ribavirin remains part of the Nipah treatment guidelines in India^79^^,^^80^ but not in Bangladesh.^81^ Consultation with Nipah stakeholders would help to establish whether the unclear effectiveness of ribavirin justifies its further use as a drug to treat Nipah or Hendra virus disease, especially with the availability of more promising alternatives.

Chloroquine did not protect ferrets^48^ or hamsters^47^ from NiV-M and was lethal at high doses in hamsters.^47^ The narrow therapeutic dose window of chloroquine is well established in clinical practice. Despite being considered a safe and effective antimalarial drug, chloroquine has been used for rapid self-poisoning in deliberate overdoses.^69^^,^^82^ Chloroquine should not be used to treat or prevent Nipah or Hendra virus disease.

The promising in-vitro efficacy of the experimental small molecules listed in table 2 is yet to be translated into convincing in-vivo protection. The parent drug of 6-azauridine (azaribine) has been withdrawn from the market due to safety concerns of thrombosis,^83^ and the parent drug of ALS-8112 (lumicitabine) has been withdrawn from development due to safety concerns of paediatric neutropenia.^84^ Whether periodate heparin, fusion lipopeptides, and defective viral particles can be manufactured at scale or are stable for stockpiling remains to be resolved.

Antivirals appear to have a narrow temporal window within which they are most likely to have clinically relevant effectiveness, thereby restricting their use to prophylaxis (pre-exposure and postexposure) and possibly, early treatment.^19^ Antivirals could also play a key role in providing bridging protection before vaccine response or availability of vaccine. The time window for protection after challenge^49^ in monkeys is shorter with NiV-B than with NiV-M,^27^ although this finding is yet to be validated in humans.

Immunomodulators could be used in combination with pathogen-directed antivirals^85^ in the later phases of infection when immunopathology is thought to dominate,^19^ although no data on such combinations are available yet. Rintatolimod is the only host-directed agent with in-vivo efficacy data specifically for henipavirus infection that has been identified in this Review, and rintatolimod provides only partial protection after low-dose NiV-M challenge in hamsters.^46^

Overall, the paucity of drug candidates and clinical evidence underscores the challenges of clinical development of therapeutics for rare but high-threat infections with the potential to cause a pandemic.

Despite further Nipah outbreaks, cases under the current epidemiological situation are insufficient to obtain phase 3 RCT efficacy data necessary for approval^86^ or to attract substantial commercial investment. Alternative approaches similar to the regionally driven end-to-end West African Lassa fever Consortium^87^ framework are needed to bridge the gap.^19^

The requirement for BSL-4 precautions for preclinical studies of Nipah or Hendra virus also restricts the studies to a small number of specialist facilities. Establishing BSL-4 facilities in a larger number of locations, especially in henipavirus-endemic areas, could increase global capacity and allow endemic countries greater freedom to prioritise studies according to local needs. However, the high operational costs, small pool of specialist staff, and shortage of non-human primates following COVID-19 remain challenges for BSL-4 studies.

In the absence of RCTs in outbreak settings, evaluation of the efficacy of therapeutics for Nipah and Hendra virus diseases is mostly reliant on controlled animal challenge studies. The variable agreement between the results for in-vitro and in-vivo efficacy for most of the small molecules identified in this Review emphasises the importance of animal efficacy data for clinical prioritisation.

The US Food and Drug Administration allows for approval of drugs for conditions that threaten global human health security under the Animal Rule,^88^ when field trials are not possible, provided the following four criteria are met: sufficient understanding of the pathophysiology of the condition and the mechanism of its reduction by the product; efficacy shown in at least two animal species or one species that is a well characterised model for predicting the product’s response in humans; an animal study endpoint that is clearly related to the desired outcome in humans, typically reduction in mortality or major morbidity; and pharmacokinetic and pharmacodynamic data from animals and humans supporting selection of an effective dose in humans. The anti-infective agents approved under the Animal Rule include raxibacumab and obitoxaximab for anthrax; antibiotics such as ciprofloxacin for plague; and tecovirimat and brincidofovir for smallpox.

The European Medicines Agency has a similar Exceptional Circumstances^89^ mechanism for granting marketing authorisation to drugs whenever collection of data on their comprehensive efficacy and safety under typical conditions of use is not possible.

The clinical and pathological features and the strengths and limitations of the major animal models of Nipah virus disease (African green monkeys,^90^ ferrets,^91^ and Syrian golden hamsters^92^) have been reviewed^93^ by the Coalition for Epidemic Preparedness Innovations (CEPI). African green monkeys are closest in physiology to humans but have less consistent neurological signs compared with hamsters and ferrets.^93^ CEPI is also improving these models, particularly by standardising the virus challenge stock.^93^ Supporting access to NiV-B strains and standardisation of the dose and route of the challenge for each model could aid comparability across studies. For challenge studies involving therapeutics, having uninfected controls to assess drug toxicity thresholds (for candidates with a narrow or unknown therapeutic dose window) and pharmacokinetic–pharmacodynamic sampling to identify in-vivo EC_50_ values and concentration–efficacy relationships would be crucial for dose optimisation to de-risk human trials. Reporting guidelines can also play an important role in supporting greater transparency and consistency in publications as well as scientific reproducibility.

Human data remain essential for the evaluation of drug safety.^88^ Before deployment in outbreak settings, phase 1 first-in-human safety data need to be collected for new therapeutic agents, and the existing experience from repurposed agents has to be critically appraised for any potential exacerbation of adverse effects by the pathophysiology of Nipah or Hendra virus infection.

In an outbreak, new therapeutic agents should be evaluated in well designed phase 2 clinical trials integrated into and sustained in health systems^94^ using preapproved standardised protocols that maximise statistical and operational efficiency in the assessment of internationally agreed-upon core outcome measures.^95^ Wherever possible, drug concentrations should be measured at the same timepoints as efficacy and safety outcomes to characterise and quantify pharmacokinetic–pharmacodynamic relationships, including those at different stages of disease.

When RCTs are not possible, observational studies using enhanced clinical characterisation protocols^96^ that incorporate the same outcome measures could provide observational data of higher quality.^97^ The long-term neurological sequelae of Nipah encephalitis^8^^,^^98^^,^^99^ also merit more systematic characterisation and potential inclusion as outcomes.

Outbreaks of high-threat infections invoke the ethical duty^100^^,^^101^ to conduct inclusive research with speed and rigour. Community and stakeholder engagement,^101^ including that for design and interventions in trials, are key to support genuine informed consent and to maintain trust in the scientific process.^102^

Selection, optimisation, and stockpiling of potential therapeutics and their appropriate dosing regimens on the basis of all available clinical and preclinical evidence in advance of any outbreak are crucial. The continuous iterative process should be guided by disease-specific and, wherever appropriate, product-specific target product profiles^103^ (comprising indication, safety, efficacy, route, stability, and affordability characteristics) developed through consensus among all relevant stakeholders, including regulators, end-users, and communities. Systems pharmacology and statistical, mathematical, and economic modelling are powerful tools to support decision making as they provide a formal framework for integration of typically sparse data from multiple study types, species, and diseases and also inform the design efficiency of phase 1 and phase 2 RCTs.

## Conclusions

At present, sufficient evidence is available to trial the monoclonal antibodies 1F5 and m102.4 along with the small molecule remdesivir (alone or in combination) for prophylaxis and early treatment of Nipah virus disease. In addition to well designed RCTs, in-vivo pharmacokinetic–pharmacodynamic studies are needed to support drug selection and dose optimisation for all high-threat infections.

## Declaration of interests

We declare no competing interests.
