# Supplementary material for: Therapeutics for Nipah virus disease: a systematic review to support prioritisation of drug candidates for clinical trials
Source: Lancet Microbe. 2025 May;6(5):None. doi: 10.1016/j.lanmic.2024.101002 (PMC12062192; doi:10.1016/j.lanmic.2024.101002)
Supplement: Supplementary appendix [file mmc1.pdf]

THE LANCET
Microbe

Supplementary appendix

This appendix formed part of the original submission and has been peer reviewed.
We post it as supplied by the authors.

Supplement to: Chan XHS, Haeusler IL, Choy BJK, et al. Therapeutics for Nipah virus disease: a systematic review to support prioritisation of drug candidates for clinical trials. *Lancet Microbe* 2025. https://doi.org/10.1016/j.lanmic.2024.101002

SUPPLEMENTARY APPENDIX

Nipah Virus Therapeutics: A Systematic Review to Support Prioritisation for Clinical Trials

Xin Hui S Chan, Ilsa L Haeusler, Bennett J K Choy, Md Zakiul Hassan, Junko Takata, Tara P Hurst, Luke M Jones, Shanghavie Loganathan, Elinor Harriss, Jake Dunning, Joel Tarning, Miles W Carroll, Peter W Horby, Piero L Olliaro

Contents

Supplementary Methods 3

Search Strategies – Bibliographic Databases 3

Search Strategies – Trial Registries 4

Search Strategies – Guidelines and Reports 4

Supplementary Results 5

Additional Text – Included Studies 5

Additional Text – Small Molecules 6

Additional Tables – Included Studies 7

Risk of Bias Assessments – Additional Figures 26

PRISMA 2020 Checklist 28

Tables

TABLE I: NIPAH & HENDRA VIRUS THERAPEUTIC MONOCLONAL ANTIBODIES (CLINICAL & ANIMAL STUDIES) 7

TABLE II: NIPAH & HENDRA VIRUS THERAPEUTIC SMALL MOLECULES (CLINICAL & ANIMAL STUDIES) 12

TABLE III: NIPAH & HENDRA VIRUS THERAPEUTIC SMALL MOLECULES (*IN VITRO* STUDIES) 17

TABLE IV: NIPAH & HENDRA VIRUS THERAPEUTIC SMALL MOLECULES (EXPLORATORY *IN VITRO* STUDIES) 21

TABLE V: NIPAH & HENDRA VIRUS THERAPEUTICS ANIMAL CHALLENGE STUDIES BY DRUG, VIRAL CHALLENGE STRAIN, AND ANIMAL MODEL 24

Figures

FIGURE I: RISK OF BIAS ASSESSMENT OF RANDOMISED CLINICAL TRIALS BY INDIVIDUAL STUDY 26

FIGURE II: SUMMARY RISK OF BIAS ASSESSMENT OF NON-RANDOMISED CLINICAL STUDIES 26

FIGURE III: RISK OF BIAS ASSESSMENT OF OBSERVATIONAL STUDIES BY INDIVIDUAL STUDY 26

FIGURE IV: SUMMARY RISK OF BIAS ASSESSMENT OF ANIMAL STUDIES 27

FIGURE V: RISK OF BIAS ASSESSMENT OF ANIMAL STUDIES BY INDIVIDUAL STUDY 27

Supplementary Methods

Search Strategies – Bibliographic Databases

PubMed

((("Henipavirus Infections"[Mesh]) OR "Henipavirus"[Mesh]) OR (henipavir*[Text Word] OR nipah*[Text Word] OR hendra*[Text Word])) AND (("Therapeutics"[Mesh]) OR "Antibodies, Monoclonal"[Mesh] OR (treat*[Text Word] OR therap*[Text Word] OR pharmacotherap*[Text Word] OR monoclonal[Text Word]))

Ovid Embase

1974 to present

1 exp henipavirus/ (1983)
2 Nipah virus infection/ (532)
3 Hendra virus infection/ (155)
4 (henipavir* or nipah* or hendra*).ti,ab,kw. (2189)
5 1 or 2 or 3 or 4 (2707)
6 exp therapy/ (11017260)
7 exp monoclonal antibody/ (833266)
8 (treat* or therap* or pharmacotherap* or monoclonal).ti,ab,kw. (12189953)
9 6 or 7 or 8 (17399177)
10 5 and 9 (1012)

Ovid CAB Abstracts

1910 to 2024 Week 35

1 exp henipavirus/ (1190)
2 (henipavir* or nipah* or hendra*).ti,ab. (1263)
3 1 or 2 (1336)
4 exp therapy/ (321663)
5 exp monoclonal antibodies/ (22927)
6 (treat* or therap* or pharmacotherap* or monoclonal).ti,ab. (2484479)
7 4 or 5 or 6 (2560950)
8 3 and 7 (264)

Ovid Global Health

1973 to 2024 Week 35

1 exp henipavirus/ (1342)
2 (henipavir* or nipah* or hendra*).ti,ab. (1349)
3 1 or 2 (1427)
4 exp therapy/ (341142)
5 exp monoclonal antibodies/ (16580)
6 (treat* or therap* or pharmacotherap* or monoclonal).ti,ab. (1263019)
7 4 or 5 or 6 (1322277)
8 3 and 7 (298)

Scopus

(TITLE-ABS-KEY (henipavir* OR nipah* OR hendra*) AND TITLE-ABS-KEY (treat* OR therap* OR pharmacotherap* OR monoclonal))

Web of Science

henipavir* OR nipah* OR hendra* (Topic) and treat* or therap* or pharmacotherap* or monoclonal (Topic)

WHO Global Index Medicus
(tw:(henipavir* or nipah* or hendra*)) AND (tw:(treat* or therap* or pharmacotherap* or monoclonal))

Search Strategies – Trial Registries

Cochrane Central Register of Controlled Trials
Issue 8 of 12, August 2024

#1	MeSH descriptor: [Henipavirus Infections] explode all trees	6
#2	MeSH descriptor: [Henipavirus] explode all trees	3
#3	(henipavir* or nipah* or hendra*):ti,ab,kw	13

Clinicaltrials.gov
Condition or disease: henipavirus or Hendra or Nipah

WHO International Clinical Trials Registry Platform
https://trialsearch.who.int/AdvSearch.aspx
Title: Nipah or Hendra or henipavirus
Recruitment status is: ALL

Search Strategies – Guidelines and Reports

TRIP Database
https://www.tripdatabase.com/Searchresult?criteria=nipah%20OR%20hendra%20OR%20henipavirus&intervention=treat*%20OR%20therap*%20OR%20monoclonal*%20OR%20pharmacotherapy&comparison=&outcome=&search_type=pico
Population: Nipah or Hendra or henipavirus
Intervention: treat* OR therap* OR monoclonal* OR pharmacotherapy

WHO website
https://www.google.com/search?q=nipah+or+Hendra+or+henipavirus+site%3A.who.int&ei=Vt2UYqToH5yVhbIPiKegkAs&ved=0ahUKEwik6-Xyv4f4AhWcSkEAHYgTCLIQ4dUDCA4&uact=5&oq=nipah+or+Hendra+or+henipavirus+site%3A.who.int&gs_lcp=Cgdnd3Mtd2l6EAM6BQghEKABOgQIIRAVSgQIQRgASgQIRhgAULUBWIYdYPgdaAFwAXgAgAGpAYgBjgySAQM4LjeYAQCgAQKgAQHAAQE&sclient=gws-wiz

Nipah or Hendra or henipavirus site:.who.int

Supplementary Results

Additional Text – Included Studies

Clinical

There was only one clinical trial, a first-in-human phase 1 study in healthy volunteers of m102.4[1], a mAb targeting the Hendra virus (HeV) envelope G glycoprotein, conducted in Australia. Of the eight reports of compassionate use for treatment or post-exposure prophylaxis during Hendra or Nipah virus outbreaks, seven were case series of fewer than 10 patients in Australia[2], India (Kerala)[3-7], and Singapore[8]. The remaining outbreak report was from the two centres where 194 of the 283 cases in the 1998 Malaysia outbreak were treated, the majority with ribavirin[9].

Two additional records of outbreaks were excluded[10,11] as the full reports on the same outbreak populations had already been included. Ribavirin was used in six[2-6,9] outbreak reports, m102.4 in one[7] single-case outbreak, and empirical treatment with broad-spectrum antimicrobials for central nervous system (ceftriaxone + aciclovir) and respiratory (clarithromycin) infection in the last[8].

Animal

Of the 26 animal studies, there were nine studies in non-human primates (African green monkeys [AGMs])[12-20], five in ferrets[21-25], and 14 in Syrian golden hamsters[15,20,26-37]. All except one involved infectious challenge with Nipah and/or Hendra virus (Supplementary Table V). Nipah virus Malaysia (NiV-M) was the most common challenge strain used in 13 studies[13,20,22,23,25,26,28,30-32,34-36], followed by nine studies using Nipah virus Bangladesh (NiV-B)[12,15-18,21,26,27,38] and five HeV[14,19,22,29,32].

The non-challenge study was of the pharmacokinetics of m102.4 in healthy ferrets[24]. All nine drug studies using an NiV-B challenge strain were published in 2016 or later. Two studies had both NiV-M and HeV-infected hamster cohorts treated with the investigational drug[22,32]. The only drug with data from both NiV-M[13,23] and NiV-B[12] infected animal cohorts was m102.4, although these were from separate studies using different animal models and inoculation doses. NiV41 and its mature form 41-6 were tested respectively in NiV-B and NiV-M infected hamsters but with different sizes of challenge inoculum[26].

Viral inoculum doses were reported as plaque forming units (PFU), median tissue culture infectious dose ($TCID_{50}$), and median lethal dose (LD_{50}). The respiratory route of inoculation was preferred in monkeys (intratracheal[12-14,16,17,20] +/- intranasal[12,15-17]), and ferrets (oronasal[23,25] or intranasal[22]). Monkeys were typically challenged with 10^5 PFU[12-14,17-19] (although one study used 10^4 PFU[15] and another 10^7 PFU[20]), and ferrets with 10^3 PFU[21-23,25]. In hamsters, intraperitoneal[26,28-32,34-36] (IP) inoculation was employed in addition to the respiratory (intranasal[20,27,36,38]) route, with a wide range of doses (10^2-10^6 PFU) used (Supplementary Table V).

All animal challenge studies reported death, and time of death, as outcome measures. The majority also reported clinical outcomes (all: signs and symptoms; AGMs only: radiological changes, blood test abnormalities) with day of onset, and a smaller majority reported pathology and virology (detection of RNA, antigen, or live virus by culture) at necropsy. A minority assessed correlation between drug concentrations and survival.

Additional Text – Small Molecules

Others

ALS-8112, parent nucleoside of lumicitabine, had low micromolar range EC_{50} values (0.3-3.08µM) in CPE inhibition and viral titre reductions assays for both NiV-M and NiV-B infected human small airway cell lines (NCI-H358 & HSAEC1-KT)[33] (Supplementary Table IV).

Additional Tables – Included Studies

Table I: Nipah & Hendra Virus Therapeutic Monoclonal Antibodies (Clinical & Animal Studies)

Drug (mechanism)	Reference	Study Design	Drug Regimen & Route & Follow-up	Efficacy	Safety
m102.4<br>(anti-HeV-G)<br><br>Developer: Uniformed Services University, USA<br><br>Funder: USA NIH	Sahay 2020[7]	Clinical: compassionate use post-exposure prophylaxis during Nipah outbreak in Kerala, India (n=1)	Not available	'Full recovery'	Not available
	Playford 2020[1]	Clinical: healthy adult volunteers (18-50 years) phase 1 dose-escalation RCT for safety, tolerability, and pharmacokinetics in Brisbane, Australia (n=40)	-Cohort 1: 1mg/kg IV day 1 (n=6)<br>-Cohort 2: 3mg/kg IV day 1 (n=6)<br>-Cohort 3: 10mg/kg IV day 1 (n=6)<br>-Cohort 4: 20mg/kg IV day 1 (n=6)<br>-Cohort 5: 20mg/kg IV day 1 & 4 (n= 6)<br>+ placebo in each cohort (n=2)<br><br>113-day follow-up (cohorts 1-4) or 123-day follow-up (cohort 5)	-PK linear<br>-Elimination kinetics of 2-dose regimen similar to 1-dose<br>-Neutralisation activity for NiV-B and HeV present in all samples at all timepoints	-No SAEs<br>-Similar rates of TEAEs between treatment and placebo groups, most commonly headache (12/30 after m102.4 vs 3/10 after placebo)<br>-No anti-m102.4 antibodies detected
	Mire 2016[12]	Animal: AGM challenge with NiV-B for efficacy and safety (n=11)<br>• 2.5×10^5 PFU intratracheal + 2.5×10^5 PFU intranasal	Treatment: ~15mg/kg IV post-challenge (n=9)<br>-Cohort 1: days 1 & 3 (n=3)<br>-Cohort 2: days 3 & 5 (n=3)<br>-Cohort 3: days 5 & 7 (n=3)<br>Control: saline (n=2)<br><br>28-day follow-up then euthanasia	**Treatment: all treated before day 5 survived to study endpoint**<br>-Cohort 1: all survived, minimal respiratory signs, normal haematology and minor biochemistry abnormalities<br>-Cohort 2: all survived, no clinical signs, mild changes in haematology and biochemistry<br>-Cohort 3: all died on day 8 with clinical and laboratory abnormalities similar to controls<br>**Controls: both died on day 7 or 8**<br>-Detectable neutralising antibody to study end in surviving animals but not deaths<br>-NiV-related gross pathological changes present in animals which died but not in surviving animals	No AEs
	Geisbert 2014[13]	Animal: AGM challenge with NiV-M for efficacy and safety (n=16)<br>• 5×10^5 PFU intratracheal	Treatment: ~15mg/kg IV post-challenge (n=12)<br>-Cohort 1: days 1 & 3 (n=4)<br>-Cohort 2: days 3 & 5 (n=4)<br>-Cohort 3: days 5 & 7 (n=4)<br>Control: saline (n=4)<br><br>28 to 34-day follow-up then euthanasia	**Treatment: all survived to study endpoint**<br>-Cohort 1: no clinical or laboratory changes<br>-Cohort 2: mild changes in haematology, biochemistry, coagulation<br>-Cohort 3: clinical signs and abnormal haematology, biochemistry, coagulation results but recovered by day 17<br>**Controls: all died between days 8 to 10**<br>-Detectable neutralising antibody to end of study in surviving animals but not in deaths<br>-NiV-related gross pathological changes present in animals which died but not in surviving animals	No AEs

	Bossart 2011[14]	Animal: AGM challenge with HeV for efficacy and safety (n=14)<br>• 4×10^5 $TCID_{50}$ intratracheal<br><br>Animal: AGM pharmacokinetics (n=4)	Efficacy<br>Treatment: 100mg IV (~25mg/kg) post-challenge<br>-Cohort 1: 10h & day 3 (n=4)<br>-Cohort 2: 24h & day 3 (n=4)<br>-Cohort 3: 72h & day 5 (n=4)<br>Control: saline (n=2)<br><br>40-day follow-up then euthanasia except for 3 animals in cohort 1 where euthanasia was on day 88<br><br>Pharmacokinetics<br>-PK 1: 10mg IV (~2.5mg/kg) (n=2)<br>-PK 2: 50mg IV (~11mg/kg) (n=2)	Efficacy<br>**Treatment: all survived to study endpoint**<br>-Cohorts 1 & 2: mild or no clinical signs of disease, no radiological changes, normal haematology and biochemistry<br>-Cohort 3: temporary moderate to severe neurological signs improved by day 16, one transient mild interstitial pneumonia on day 6, transient fall in platelet count days 6-13<br>**Controls: both died after average of 8 days**<br>-m102.4 concentrations on day 3 correlated with survival.<br>-HeV-related gross pathological changes present in animals which died but not in surviving animals.<br>Pharmacokinetics<br>Average distribution and elimination half-lives of ~1 day and ~11 days respectively	No AEs
	Bossart 2009[23]	Animal: Ferret challenge with NiV-M for efficacy (n=8)<br>• 5×10^3 $TCID_{50}$ oronasal	Treatment: 50mg IV (n=6)<br>-Cohort 1: 24h pre-challenge (n=3)<br>-Cohort 2: 10h post-challenge (n=3)<br>Control: PBS (n=2)<br>-Control 1: 24h pre-challenge (n=1)<br>-Control 2: 10h post-challenge (n=1)<br><br>20-day follow-up then euthanasia	**Treatment: all survived to study endpoint if treated 10h post-challenge but not 24h pre-challenge**<br>-Cohort 1: 2/3 died on day 13 after all developed severe disease from day 7<br>-Cohort 2: all survived but had clinical symptoms from day 8<br>**Controls: both died on day 8 after becoming unwell on day 6**<br>-m102.4 concentrations on day 3 correlated with survival.<br>-NiV-related gross pathological changes present in animals which died but not in surviving animals.<br>-Neutralisation activity for NiV-B, NiV-M, and HeV present.	Not available
	Zhu 2008[24]	Animal: Ferret pharmacokinetics (n=4)	-Cohort 1: 5mg IV (n=2)<br>-Cohort 2: 25mg IV (n=2)<br>42-day follow-up then euthanasia	-Average distribution and elimination half-lives 1.48 and 3.58 days respectively for both doses with small inter-individual differences<br>-Neutralisation activity for NiV present for 8 days	-No AEs<br>-No anti-m102.4 antibodies found
1F5 (anti-NiV-F) vs m102.4 (anti-HeV-G)	Zeitlin 2024[15]	Animal: AGM challenge with NiV-B for efficacy (n=13)<br>• 4×10^4 PFU intranasal	Treatment 1: IV 5 days post-challenge (n=12)<br>-Cohort 1: 1F5 25mg/kg (n=6)<br>-Cohort 2: m102.4 25mg/kg (n=6)<br>Control 1: untreated infected (n=1)<br>Treatment 2: IV 5 days post-challenge (n=3)<br>-Cohort 3: 1F5 10mg/kg (n=3)<br>Control 2: untreated infected (n=1)<br>35-day follow-up then euthanasia	**Treatments 1 & 2: 1F5 not m102.4 provided complete protection**<br>-Cohort 1: all survived to study endpoint with minimal clinical signs<br>-Cohort 2: 1/6 survived to study endpoint<br>-Cohort 3: all survived to study endpoint with minimal clinical signs<br>**Controls 1 & 2: both were euthanised for disease severity on days 7 and 9 respectively**	Not available
1F5 vs 12B2 vs 1F5 + 12B2 (anti-NIV-F)<br><br>Developer: Uniformed Services University, USA<br><br>Funder: USA NIH		Animal: Hamster challenge with NiV-B for efficacy (n=16)<br>• 5×10^6 PFU intranasal	Treatment 3: IP 24h post-challenge (n=15)<br>-Cohort 4: 1F5 5mg/kg (n=5)<br>-Cohort 5: 12B2 5mg/kg (n=5)<br>-Cohort 6: 1F5 2.5mg/kg + 12B2 2.5mg/kg (n=5)<br>Control 3: PBS (n=1)<br>28-day follow-up then euthanasia	**Treatment 3: 1F5 not 12B2 provided complete protection**<br>-Cohort 4: all survived to study endpoint<br>-Cohort 5: 3/5 survived to study endpoint<br>-Cohort 6: 4/5 survived to study endpoint<br>**Control 3: died on day 8 post-challenge**	

h5B3.1 (anti-NiV-F)<br><br>Developer: Uniformed Services University, USA<br><br>Funder: USA NIH	Mire 2020[22]	Animal: Ferret challenge with NiV-M or HeV for efficacy (n=11)<br>• 5 x 10^3 PFU intranasal	Treatment: 20mg/kg IP post-challenge<br>NiV-M<br>-Cohort 1: days 1 & 3 (n=3)<br>-Cohort 2: days 3 & 5(n=3)<br>Control 1: untreated infected (n=1)<br>HeV<br>-Cohort 3: days 3 & 5 (n=3)<br>Control 2: untreated infected (n=1)<br>34-day follow-up then euthanasia	**Treatment: h5B3 conferred complete protection to study endpoint**<br>NiV-M<br>All survived after minor clinical signs and gained weight<br>HeV<br>All survived after minor clinical signs and gained weight<br>**Controls: both died on days 8-9**	Not available
NiV41<br>(anti-NiV-RBP)	Chen 2024[26]	Animal: Hamster challenge with NiV-B for efficacy (n=12)<br>• 10^5 $TCID_{50}$ intraperitoneal	Treatment 1: 3mg/kg IP 6 hours post-challenge (n=6)<br>Control: PBS (n=6)<br>28-day follow-up then euthanasia	**Treatment 1: NiV41 gave complete protection to study endpoint**<br>**Controls: all except two were euthanised for disease severity**	Not available
NiV41-6<br>(anti-NiV-RBP)<br><br>Developer: Wuhan Institute of Virology<br><br>Funder: Chinese Academy of Sciences		Animal: Hamster challenge with NiV-M for efficacy (n=48)<br>• 1000 LD_{50} intraperitoneal	Prophylaxis: IP 24 hours pre-challenge (n=12)<br>-Cohort 1: 10mg/kg (n=6)<br>-Cohort 2: 3mg/kg (n=6)<br>Controls 1: PBS (n=6 for each cohort volume)<br><br>Treatment 2: 10mg/kg IP post-challenge (n=18)<br>-Cohort 3: 3 hours (n=6)<br>-Cohort 4: 3 hours & 3 days (n=6)<br>-Cohort 5: 1 day & 3 days (n=6)<br>Controls 2: PBS (n=6)<br><br>28-day follow-up then euthanasia	**Prophylaxis: NiV41-6 dose-dependent protection**<br>-Cohort 1: all survived to study endpoint<br>-Cohort 2: 5/6 survived to study endpoint<br>**Controls 1: all except one were euthanised for disease severity**<br><br>**Treatment 2: NiV41-6 administration time-dependent protection**<br>-Cohort 3: 5/6 survived to study endpoint<br>-Cohort 4: 4/6 survived to study endpoint<br>-Cohort 5: 3/6 survived to study endpoint<br>**Controls 2: all except one were euthanised for disease severity**	
HENV-103, HENV-117, HENV-58, HENV-98, HENV-100 (anti-HeV-RBP)<br><br>Developer: Vanderbilt University, USA<br><br>Funder: USA NIH	Doyle 2021[27]	Animal: Hamster challenge with NiV-B for efficacy (n=46)<br>• 5 x 10^6 PFU intranasal	Treatment 1: 10mg/kg IP 24h post-challenge (n=25)<br>-Cohort 1: HENV-103 (n=5)<br>-Cohort 2: HENV-117 (n=5)<br>-Cohort 3: HENV-58 (n=5)<br>-Cohort 4: HENV-98 (n=5)<br>-Cohort 5: HENV-100 (n=5)<br>Control 1: untreated infected (n=1)<br><br>Treatment 2: 10mg/kg IP 24h post-challenge (n=15)<br>-Cohort 6: HENV-103 + HENV-117 5mg/kg each (n=5)<br>-Cohort 7: HENV-117-103 DVD (n=5)<br>-Cohort 8: HENV-117-103 Bis4Ab (n=5)<br>Control 2: PBS (n=5)<br><br>28-day follow-up then euthanasia	**Treatment 1: partial protection from individual mAbs**<br>-Cohorts 1 & 3: 2/5 survived to study endpoint<br>-Cohort 2, 4 & 5: 3/5 survived to study endpoint<br>**Control 1: died on day 3**<br><br>**Treatment 2: complete protection after mAb cocktail but partial protection from bispecific mAbs**<br>-Cohort 6: all survived to study endpoint<br>-Cohort 7: 4/5 survived to study endpoint<br>-Cohort 8: 3/5 survived to study endpoint<br>**Control 2: 4/5 died**	Not available
HENV-26, HENV-32 (anti-HeV-RBP)	Dong 2020[21]	Animal: Ferret challenge with NiV-B for efficacy (n=13)<br>• 5 x 10^3 PFU intranasal	Treatment: 15mg/kg IP days 3 & 5 post-challenge (n=10)<br>-Cohort 1: HENV-26 (n=5)	**Treatment: HENV-26 & HENV-32 conferred complete protection**<br>-Cohort 1: no clinical disease, transient haematological changes, no detectable viral genomes in blood	Not available

Developer:<br>Vanderbilt University, USA<br><br>Funder: USA NIH			-Cohort 2: HENV-32 (n=5)<br>Control: untreated infected (n=3)<br><br>28-day follow-up then euthanasia	-Cohort 2: 4/5 developed clinical disease with respiratory signs, viral genomes detected in blood on day 5 (3/5) and day 14 (1/5)<br>**Controls: all died between days 7-8**<br>-NiV-related gross pathological changes present in animals which died but not in surviving animals	
NipGIP1.7 & Nip3B10 (anti-NiV-G), NipGIP35 & NipGIP3 (anti-NiV-F)<br><br>Developer:<br>INSERM, France<br><br>Funders:<br>Aventis Pharma, Bayer Pharma, INSERM & Institut Pasteur	Guillaume 2006[28]	Animal: Hamster challenge with NiV-M for efficacy, dose titration, and therapeutic time window (n=124)<br>• 7.5 x 10^2 PFU (100 LD_{50}) intraperitoneal	<u>Protection</u><br>Treatment 1: 24h pre- & 1h post-challenge IP (n=32)<br>-Cohort 1: NipGIP1.7 112µg (n=8)<br>-Cohort 2: Nip3B10 100µg (n=8)<br>-Cohort 3: NipGIP35 180µg (n=8)<br>-Cohort 4: NipGIP3 520µg (n=8)<br>Control 1: no mAb (n=8)<br>65-day follow-up<br><br><u>Dose Titration</u><br>Treatment 2: 24h pre- & 1h post-challenge IP (n=40)<br>-Cohort 5: NipGIP1.7 112µg (n=4)<br>-Cohort 6: NipGIP1.7 1.12µg (n=4)<br>-Cohort 7: NipGIP1.7 0.12µg (n=4)<br>-Cohort 8: NipGIP1.7 0.012µg (n=4)<br>-Cohort 9: NipGIP1.7 0.0012µg (n=4)<br>-Cohort 10: NipGIP35 180µg (n=4)<br>-Cohort 11: NipGIP35 1.8µg (n=4)<br>-Cohort 12: NipGIP35 0.18µg (n=4)<br>-Cohort 13: NipGIP35 0.018µg (n=4)<br>-Cohort 14: NipGIP35 0.0018µg (n=4)<br>Control 2: no mAb (n=4)<br>36-day follow-up then euthanasia<br><br><u>Therapeutic Time Window</u><br>Treatment 3: NipGIP1.7 112µg IP (n=20)<br>-Cohort 15: 1h post-challenge (n=4)<br>-Cohort 16: 24h post-challenge (n=4)<br>-Cohort 17: 48h post-challenge (n=4)<br>-Cohort 18: 72h post-challenge (n=4)<br>-Cohort 19: 96h post-challenge (n=4)<br>Treatment 4: NipGIP35 180µg IP (n=20)<br>-Cohort 20: 1h post-challenge (n=4)<br>-Cohort 21: 24h post-challenge (n=4)<br>-Cohort 22: 48h post-challenge (n=4)<br>-Cohort 23: 72h post-challenge (n=4)<br>-Cohort 24: 96h post-challenge (n=4)<br>86-day follow-up	<u>Protection</u><br>**Treatment 1: 30/32 treated survived to study endpoint**<br>-Cohorts 1-3: all survived to study endpoint<br>-Cohort 4: 6/8 survived to study endpoint<br>**Controls 1: all died**<br><br><u>Dose Titration</u><br>**Treatment 2: survival is mAb dose-dependent**<br>-Cohorts 5 & 6: all survived to study endpoint<br>-Cohorts 7-9: 1/4 survived to study endpoint<br>-Cohort 10: all survived to study endpoint<br>-Cohort 11: 2/4 survived to study endpoint<br>-Cohorts 12-14 & control 2: all died<br><br><u>Therapeutic Time Window</u><br>**Treatment 3: survival is mAb administration time-dependent**<br>-Cohort 15: 3/4 survived to study endpoint<br>-Cohort 16: 2/4 survived to study endpoint<br>-Cohorts 17-19: all died to study endpoint<br>-Cohort 20: all survived to study endpoint<br>-Cohorts 21-22: 2/4 survived to study endpoint<br>-Cohort 23: 1/4 survived to study endpoint<br>-Cohort 24: 2/4 survived to study endpoint	Not available

NipGIP35, NipGIP3, NipGIP21, NipGIP7 (anti-NiV-F)<br><br>Institution:<br>INSERM, France<br><br>Funders:<br>Aventis Pharma, Bayer Pharma, INSERM & Institut Pasteur	Guillaume 2009[29]	Animal: Hamster challenge with HeV for efficacy and dose titration (n=54)<br>• 10^3 PFU (100 LD_{50}) intraperitoneal	<u>Protection</u><br>Treatment 1: 24h pre- & 1h post-challenge IP (n=24)<br>-Cohort 1: 2.5mg/kg NipGIP35 (n=6)<br>-Cohort 2: 6mg/kg NipGIP3 (n=6)<br>-Cohort 3: 2.7mg/kg NipGIP7 (n=6)<br>-Cohort 4: 4.2mg/kg NipGIP21 (n=6)<br>Control 1: PBS (n=6)<br>30-day follow-up<br><br><u>Dose Titration</u><br>Treatment 2: 1h pre-challenge IP (n=18)<br>-Cohort 5: 3mg/kg NipGIP21 (n=6)<br>-Cohort 6: 0.3mg/kg NipGIP21 (n=6)<br>-Cohort 7: 0.03mg/kg NipGIP21 (n=6)<br>Control 2: PBS (n=6)<br>14-day follow-up	<u>Protection</u><br>**Treatment 1: all treated survived to study endpoint**<br>**Controls 1: all died within 7 days**<br><br><u>Dose Titration</u><br>**Treatment 2: survival is mAb dose-dependent**<br>-Cohort 5: 5/6 survived to study endpoint<br>-Cohort 6: 3/6 survived to study endpoint<br>-Cohort 7: 2/6 survived to study endpoint<br>Controls 2: 5/6 died	Not available

AE = adverse event; AGM = African Green monkey; DVD = dual variable domain; HeV = Hendra virus; INSERM = Institut National de la Santé et de la Recherche Médicale; IP = intraperitoneal; IV = intravenous; LD_{50} = median lethal dose; mAb = monoclonal antibody; NIH = National Institutes of Health; NiV-B = Nipah virus Bangladesh; NiV-M = Nipah virus Malaysia; PBS = phosphate-buffered saline; PFU = plaque-forming units; PK = pharmacokinetics; RBP = receptor binding protein; RCT = randomised controlled trial; SAE = serious adverse event; $TCID_{50}$ = median tissue culture infectious dose; TEAE = treatment emergent adverse event; USA = United States of America

Table II: Nipah & Hendra Virus Therapeutic Small Molecules (Clinical & Animal Studies)

Drug (mechanism)	Reference	Study Design	Drug Regimen & Route & Follow-up	Efficacy	Safety
Ribavirin (nucleoside analogue prodrug)	Warrier 2020[6]	Clinical: compassionate use for treatment in Nipah outbreak in Kochi, India, 2019 (n=1)	Not available<br>Also treated with immunoglobulins	Survived and recovered fully from encephalitis after 51 days	Not available
	Radhakrish-nan 2020[5]	Clinical: compassionate use for treatment in Nipah outbreak in Kerala, India, 2018 (n=12: 6 treated, 6 untreated)	2g IV loading followed by 1g IV QDS for 4 days then 500mg PO QDS for 6 days	Treated group: 4/6 died<br>Untreated group: 6/6 died	Not available
	Banerjee 2019[3]	Clinical: compassionate use for post-exposure prophylaxis of healthcare workers during Nipah outbreak in Kerala, India, 2018 (n=8)	1g TDS for 14 days administered within 72 hours from exposure. Route not available.	None developed Nipah infection	None completed course<br>-6/8 had transient increase in bilirubin and/or fall in haemoglobin levels<br>-6/8 experienced symptoms of fatigue, headache, nausea, dry mouth, and palpitations
	Kumar 2019[4]	Clinical: compassionate use for treatment in Nipah outbreak in Kerala, India, 2018 (n=5)	Not available	All died	Not available
	Playford 2010[2]	Clinical: compassionate use during Hendra outbreak in Australia, 2008 for treatment (n=2) and post-exposure prophylaxis (n=1)	Treatment:<br>-Patient 1: 30mg/kg IV loading, then 15mg/kg IV QDS for 4 days, then 8mg/kg IV TDS for 12 days<br>-Patient 2: 30mg/kg IV loading, then 15mg/kg IV QDS for 32 days, then 600mg PO TDS for until month 8<br>Prophylaxis:<br>30mg/kg IV loading, then 15mg/kg IV QDS for 5 days within 4 hours from exposure	-Patient 1 died while patient 2 made a full recovery from encephalitis<br>-Contact did not seroconvert	-Ribavirin stopped in patient 1 after 12 days due to development of anaemia (Hb 76 g/L)<br>-Well-tolerated by other recipients
	Chong 2001[9]	Clinical: compassionate use for treatment in Nipah outbreak in Malaysia, 1998-99 (n=194: 140 treated, 54 untreated)	IV (n=128):<br>30mg/kg loading, then 16mg/kg QDS for 4 days, then 8 mg/kg TDS for 3 days<br>PO (n=12):<br>2g on day 1, 1.2g TDS on days 2-4, 1.2g BD on days 5-6, 0.6g BD for another 1 to 4 days	Treatment group: 32% died (45/140)<br>Non-treatment group: 54% died (29/54)	No statistically significant difference in incidence of anaemia and bilirubinaemia in both groups
	Rockx 2010[19]	Animal: AGM challenge with HeV (n=12)<br>• 4 x 10^5 $TCID_{50}$ intratracheal for efficacy	Treatment: 50mg/kg SC loading, then 10mg/kg SC TDS for 14 days (n=9)<br>-Cohort 1: 24 hours pre-challenge (n=3)<br>-Cohort 2: 12 hours post-challenge (n=3)<br>-Cohort 3: 48 hours post-challenge (n=3)<br>Control: PBS (n=3)<br>14-day follow up	Cohorts 1 & 2: symptom onset on days 5-9, time to death 8.5-10.5 days, shift from primarily respiratory to neurological signs<br>Cohort 3 & control: symptom onset on days 5-6, time to death 7-9 days<br>-NiV-related radiological and gross pathological changes more severe in cohort 3 & controls than cohorts 1 & 2<br>-Reduction in infectious virus titres in cohorts 1-3 and number of virus-positive tissues in cohort 1 but not controls	Not available

Ribavirin (nucleoside analogue prodrug) & 6-azauridine (OMP decarboxylase inhibitor) & Rintatolimod (TLR-3 agonist interferon inducer)	Georges-Courbot 2006[31]	Experiment 1<br>Animal: Hamster challenge with NiV-M for efficacy (n=18)<br>• 350 x LD_{50} intraperitoneal<br><br>Experiment 2<br>Animal: Hamster challenge with NiV-M for efficacy (n=18)<br>• 35 x LD_{50} intraperitoneal	Experiment 1<br>Treatment 1: SC continuous infusion via osmotic pump from immediately prior to challenge for 14 days<br>-Cohort 1: ribavirin 50mg/kg/day (n=6)<br>-Cohort 2: 6-aza-uridine 175mg/kg/day (n=6)<br>Control 1: PBS (n=6)<br><br>14-day follow up<br><br>Experiment 2<br>Treatment 2: IP from 2 hours post challenge for 10 days<br>-Cohort 3: ribavirin 25mg/kg BD (n=6)<br>-Cohort 4: rintatolimod 3mg/kg OD (n=6)<br>Control 2: PBS (n=6)<br><br>30-day follow up then euthanasia	Experiment 1<br>All died but ribavirin and 6-aza-uridine delayed mean time to death<br>-Cohort 1: 6.8 ± 0.7 days (p<0.01)<br>-Cohort 2: 6.1 ± 0.7 days (p<0.05)<br>Control 1: 5.1 ± 0.7 days<br><br>-Viral RNA detected in all tissues from all groups tested<br><br>Experiment 2<br>Partial protection from rintatolimod<br>-Cohort 3: 1/6 survived<br>-Cohort 4: 5/6 survived, no infectious virus detected in surviving animals, infectious virus and viral RNA detected in brain of animal which died<br>Control 2: 1/6 survived	Not available
Ribavirin (nucleoside analogue prodrug) & chloroquine (lysosome alkalinisation)<br><br>Funder: USA NIH	Freiberg 2010[32]	Animal: Hamster challenge with NiV-M (n=41) and HeV (n=20) for efficacy (n=85)<br>• 10^4 $TCID_{50}$ intraperitoneal	Experiment 1<br>Treatment 1: IP from 6 hours post-challenge with NiV-M (n=15) or HeV (n=15) for 21 days<br>-Cohort 1 & 4: ribavirin 30mg/kg BD (n=5)<br>-Cohort 2 & 5: chloroquine 50mg/kg alternate days (n=5)<br>-Cohort 3 & 6: ribavirin 30mg/kg BD + chloroquine 50mg/kg alternate days (n=5)<br>Controls 1: (n=16)<br>-Untreated: vehicle solution (n=5 for each virus)<br>-Uninfected: drugs only (n=2 per drug regimen)<br>21-day follow up<br><br>Experiment 2<br>Treatment 2: IP from 6 hours post-challenge with NiV-M only for 9 days (n=18)<br>-Cohort 7: ribavirin 50mg/kg BD (n=3)<br>-Cohort 8: ribavirin 75mg/kg BD (n=3)<br>-Cohort 9: ribavirin 100mg/kg BD (n=3)<br>-Cohort 10: chloroquine 50mg/kg OD (n=3)<br>-Cohort 11: chloroquine 100mg/kg OD (n=3)<br>-Cohort 12: chloroquine 150mg/kg OD (n=3)<br>Controls 2: (n=21)<br>-Untreated: vehicle solution (n=3)<br>-Uninfected: drug only (n=3 per drug regimen)<br>9-day follow-up	Experiment 1<br>Ribavirin alone delayed death from NiV-M<br>-Cohorts 1 & 4: Died 5 days later (NIV-M, 2 survived) or at the same time after challenge (HeV) as untreated controls<br>-Cohort 2 & 5: All died 3 days (NIV-M) or 2 days (HeV) earlier than untreated controls<br>-Cohort 3 & 6: As untreated controls<br>Controls: All untreated died on days 5-8 (NiV-M; 1 survived) and day 4 (HeV; 1 survived to day 14), all uninfected lived<br><br>Experiment 2<br>Ribavirin delayed mean time to death after NiV-M but was toxic at higher doses<br>-Cohort 7: All died 2-3 days later than infected controls<br>-Cohort 8: All died 1 day later than infected controls<br>-Cohort 9: 2/3 euthanised for drug toxicity<br>Chloroquine was lethal at higher doses<br>-Cohort 10: Course as infected controls<br>-Cohort 11 & 12: Died after 1-2 days from drug toxicity<br>Controls: All untreated died after 5 days	Not available<br><br>Higher treatment doses caused severe toxicity<br>-After ribavirin at 100mg/kg BD, all animals lost weight from days 3-4, 2/3 became unwell on day 6 requiring euthanasia<br>-After chloroquine at 100 or 150 mg/kg OD, all animals died on days 1-2 with and on day 2 without challenge
Chloroquine (lysosome	Pallister 2009[25]	Animal: Ferret challenge with NiV-M (n=8) for efficacy and pharmacokinetics	Treatment: 25mg/kg IV OD<br>-Cohort 1: 24 hours pre-challenge (n=3)	All animals became febrile with neurological symptoms and died by days 7-8. No clinical, pathological, or virological	Not available

alkalinisation)<br>Funder: USA NIH		• 5 x 10^3 $TCID_{50}$ oronasal	-Cohort 2: 10 hours post-challenge (n=3)<br>Controls: 20% sucrose (n=1 per cohort)	differences between treatment and control animals.	
Remdesivir (nucleoside analogue)<br>Developer: Gilead<br>Funder: USA NIH	de Wit 2023[16]	Animal: AGM challenge with NIV-B for efficacy (n=18)<br>• 10^5 $TCID_{50}$ intranasal + 10^5 $TCID_{50}$ intratracheal	Treatment: IV infusion from 3 days post-challenge (n=12)<br>-Cohort 1: 10mg/kg for 12 days (n=6)<br>-Cohort 2: 10mg/kg for 1 day then 5mg/kg for 11 days (n=6)<br>Controls: vehicle (n=3 for each of the two cohort volumes)<br><br>42-day follow up then euthanasia	Cohort 1: 4/6 survived, 2 euthanised between days 6-8 after developing neurological signs<br>Cohort 2: 2/6 survived, 4 euthanised between days 7-9 after developing neurological and respiratory signs<br>Controls: all euthanised between days 7-9 after all developing severe respiratory disease	Not available
	Lo 2019[17]	Animal: AGM challenge with NiV-B for efficacy (n=8)<br>• 10^5 $TCID_{50}$ intranasal + 10^5 $TCID_{50}$ intratracheal	Treatment: 10mg/kg IV OD from 1 day post-challenge for 12 days (n=4)<br>Control: vehicle solution (n=4)<br><br>92-day follow up then euthanasia	Treatment: all survived, 2/4 developed mild respiratory signs which resolved by days 12-14, none viraemic but 1/4 had detectable viral RNA in brain tissue with focal meningo-encephalitis on histology and high virus neutralising antibody titres<br>Controls: all died by day 8 after developing respiratory signs from days 3-4, all viraemic with high virus titres in all tissues	Not available
	Jordan 2017[18]	Animal: AGM challenge with NiV-B for efficacy<br>• Lethal dose (unspecified)	Treatment: 10mg/kg IV OD from 1 day post-challenge<br>35-day follow up (n=unspecified)	All animals survived with no major respiratory or CNS symptoms	Not available
Favipiravir (nucleoside analogue prodrug)<br>Developer: Toyama<br>Funder: USA NIH	Dawes 2018[30]	Animal: Hamster challenge with NiV-M for efficacy (n=18)<br>• 10^4 PFU intraperitoneal	Treatment: 600mg/kg SC immediately post-challenge; then maintenance for 13 days<br>-Cohort 1: 300mg/kg PO BD (n=5)<br>-Cohort 2: 300mg/kg SC OD (n=5)<br>Control: vehicle solution (n=4 per cohort)<br>42-day follow up then euthanasia	Treatment: all animals survived without clinical signs and gained weight<br>Controls: all died by days 5-6 after developing respiratory and neurological symptoms with severe weight loss<br>-NiV-related pathological changes and viral antigen present in animals which died but not in surviving animals	Not available
Griffithsin (GRFT) (fusion and cell entry inhibitor)<br>Funder: USA NIH & USA CDC	Lo 2020[38]	Animals: Hamster challenge with NiV-B for efficacy (n=65)<br>• 10^7 $TCID_{50}$ intranasal	Treatment 1: 10 mg/kg intranasal OD oxidation resistant GRFT (Q-GRFT)<br>-Cohort 1: days 1 & 2 pre-challenge (n=10)<br>-Cohort 2: days 1 & 2 pre-challenge then days 1 and 2 post-challenge (n=10)<br>Treatment 2: 10mg/kg intranasal OD trimeric monomer of GRFT (3mG)<br>-Cohort 1: days 1 & 2 pre-challenge (n=10)<br>-Cohort 2: days 1 & 2 pre-challenge then days 1 & 2 post-challenge (n=10)<br>Controls:<br>-Infected untreated: PBS OD days 1 & 2 pre-challenge then days 1 & 2 post-challenge (n=10)<br>-Uninfected treated: drug only (n=5 per drug)<br>-Uninfected untreated: no drug or virus (n=5)<br>28-day follow-up then euthanasia	Treatment 1 (Q-GRFT):<br>-Cohorts 1 & 2: 7/20 survived with no clear difference between cohorts, 70% of survivors had no clinical signs<br>Treatment 2 (3-mG):<br>-Cohorts 3 & 4: 3/20 survived with no clear difference between cohorts, 33% survivors had no clinical signs<br>Controls:<br>-Infected untreated: all died<br>-Uninfected treated: all survived<br>-Uninfected untreated: all survived<br><br>-NiV RNA detected in most tissues from dead/euthanised animals but only in eyes and brains of surviving treated animals	Not available
Periodate heparin (competitive	Mathieu 2015[34]	Animal: Hamster challenge with NiV-M for efficacy (n=15)	Treatment: 10mg/kg SC OD for 12 days from challenge (n=5)<br>Controls:	Treatment: 1/5 survived to day 21<br>-Untreated: all died by day 6	Not available

inhibitor of *trans*-infection)<br>Funder: INSERM		• 500 x LD_{50} intraperitoneal	-Untreated: challenge only (n=5)<br>-Uninfected: drug only (n=5)<br>21-day follow up	-Uninfected: all survived	
Fusion inhibitory lipopeptides (fusion and cell entry inhibitors):<br><br>VIKI-dPEG4-Chol, VIKI-dPEG4-Toco	Mathieu 2018[20]	Animal: Hamster challenge with NiV-M for efficacy (n=38)<br>• 10^6 PFU (100 x LD_{50}) intranasal<br><br>Animal: AGM challenge with NiV-M for efficacy (n=10)<br>• 2 x 10^7 PFU intratracheal<br><br>Animal: AGM biodistribution (n=4)	<u>Hamster</u><br>Treatment 1: 10mg/kg intranasal OD day -1 to 1 post-challenge<br>-Cohort 1: VIKI-dPEG4-Chol (n=12)<br>-Cohort 2: VIKI-dPEG4-Toco (n=6)<br>Controls 1:<br>-Untreated: vehicle control (n=12)<br>-Uninfected: drug only (n=8)<br>21-day follow up<br><br><u>Monkey</u><br>Treatment 2: VIKI-dPEG4-Toco OD<br>-Cohort 3: 10mg/kg intratracheal days -1 to 5 post-challenge (n=3)<br>-Cohort 4: 10mg/kg intratracheal days -1 to 5 + 2mg/kg SC days -1 to 10 post-challenge (n=3)<br>Controls 2:<br>-Untreated: vehicle control (n=3)<br>-Uninfected: drug intratracheal + SC only (n=1)<br>28-day follow up<br><br>Biodistribution: VIKI-dPEG4-Toco days 0 & 14<br>-Cohort 5: 10mg/kg intratracheal (n=2)<br>-Cohort 6: 10mg/kg intratracheal + 2mg/kg SC (n=2)	<u>Hamster</u><br>Treatment 1:<br>-Cohort 1: 5/12 survived to day 21<br>-Cohort 2: 3/6 survived to day 21<br>Controls 1:<br>-Untreated: all died by day 13<br>-Uninfected: all survived to day 21<br><br><u>Monkey</u><br>Treatment 2:<br>-Cohort 3: 1/3 survived<br>-Cohort 4: 1/3 survived<br>Controls 2:<br>-Untreated: all died by day 13<br>-Uninfected: all survived<br><br>Biodistribution:<br>-Intratracheal only: serum levels peaked at 200nM 4 hours after administration, undetectable at 24 hours<br>-Intratracheal + SC: serum detection at 8 hours, peaking at 500nM, <300nM at 24 hours; organ detection in brain (10nM) and lung (30-200nM) at 24 hours	<u>Monkey</u><br>VIKI-dPEG4-Toco well-tolerated with no significant adverse effects
VG-PEG24-Chol	Mathieu 2017[35]	Animal: Hamster challenge with NiV-M for efficacy (n=13)<br>• 100 x LD_{50} intraperitoneal<br><br>Animal: Hamster biodistribution (n=6)	Treatment: 2mg/kg IP OD days -1 to 10 (n=6)<br>Controls:<br>-Untreated: vehicle control (n=6)<br>-Uninfected: peptide only (n=1)<br>21-day follow up<br><br>Hamster biodistribution: 2mg/kg IP	Treatment: 5/6 survived<br>Controls: untreated all died by day 8, uninfected survived<br><br>Hamster biodistribution: free peptide in serum at 8 hours, peaking at 120nM, dropping after 24h, with peptide detection at 24h in organs including brain	Not available
VIKI-PEG4-chol<br><br>Funder: USA NIH & INSERM	Porotto 2010[37]	Animal: Hamster challenge with NiV (strain unspecified) for efficacy (n=35)<br>• 100 x LD_{50} intraperitoneal	Treatment: 2mg/kg IP OD for 14 days starting on different days relative to challenge<br>-Cohort 1: day -2 (n=5)<br>-Cohort 2: day -1 (n=5)<br>-Cohort 3: day 0 (n=5)<br>-Cohort 4: day 1 (n=5)<br>-Cohort 5: day 2 (n=5)<br>-Cohort 6: day 4 (n=5)	Treatment:<br>-Cohort 1: 4/5 survived<br>-Cohort 2: 3/5 survived<br>-Cohort 3: 4/5 survived<br>-Cohort 4: all died<br>-Cohort 5: 2/5 survived<br>-Cohort 6: 1/5 survived<br>Control: all died by day 7	Not available

			Control: vehicle solution (n=5)<br>30-day follow up		
Defective interfering particles (virus-like particles containing defective interfering genomes which inhibit replication): DI-07, DI-10, DI-14, DI-35<br><br>Funder: USA CDC	Welch 2022[36]	Animal: Hamster challenge with NiV-M for efficacy (n=153)<br>• Experiment 1: 10^4 $TCID_{50}$ intraperitoneal<br>• Experiment 2: 10^6 $TCID_{50}$ intranasal	Experiment 1 (n=99)<br>Treatment 1: 2 x 10^9 TIPs IP with challenge<br>-Cohort 1: active TIPs (n=39 in total)<br>--DI-07, DI-10, DI-35 (n=10 each); DI-14 (n=9)<br>-Cohort 2: inactive TIPs (n=40 in total)<br>--DI-07, DI-10, DI-35, DI-14 (n=10 each)<br>Controls 1: vehicle solution (n=20)<br><br>Experiment 2 (n=54)<br>Treatment 2: 1 x 10^8 active TIPs intranasal with challenge<br>-Cohort 3: active TIPs (n=34 in total)<br>--DI-07 (n=10); DI-10, DI-14, DI-35 (n=8 each)<br>Controls 2: vehicle solution (n=20)	Experiment 1<br>-Cohort 1: 11/39 survived, 17/39 had no clinical signs, surviving animals had 4.8 days of clinical signs<br>-Cohort 2: 12/40 survived, disease course similar to controls, surviving animals had 7.7 days of clinical signs<br>Controls 1: 18/20 died, surviving animals had 14 days of clinical signs<br><br>Experiment 2<br>-Cohort 3: 14/34 survived following 6.1 days of clinical signs<br>Controls 2: 5/20 survived following 13.4 days of clinical signs	Not available
Ceftriaxone (bacterial cell wall synthesis inhibitor), clarithromycin (bacterial protein synthesis inhibitor), aciclovir (nucleoside analogue)	Paton 1999[8]	Clinical: empirical syndromic treatment during outbreak in Singapore, 1999 (n=11)	Ceftriaxone + aciclovir IV (n=9 encephalitis)<br>Clarithromycin (n=2 atypical pneumonia)	Ceftriaxone + aciclovir: 8/9 survived, 4/9 had persistent neurological deficits<br>Clarithromycin: 2/2 survived	Not available

AGM = African Green monkey; CDC = Centres for Disease Control; CSF = cerebrospinal fluid; HeV = Hendra virus; dPEG = discrete Polyethylene Glycol; INSERM = Institut National de la Santé et de la Recherche Médicale; IP = intraperitoneal; IV = intravenous; LD_{50} = median lethal dose; NIH = National Institutes of Health; NiV-B = Nipah virus Bangladesh; NiV-M = Nipah virus Malaysia; nM = nanomoles; OMP = orotidine monophosphate; PBS = phosphate-buffered saline; PFU = plaque-forming units; PO = orally (per os); RNA = ribonucleic acid; SC = subcutaneous; $TCID_{50}$ = median tissue culture infectious dose; TIP = therapeutic infectious particle; TLR-3 = toll-like receptor 3; USA = United States of America

Table III: Nipah & Hendra Virus Therapeutic Small Molecules (*In Vitro* Studies)

Reference	Drug (Other Names)	Drug Type (Target)	Assays	Cells	Drug Sub-type	Viruses	EC_{50}	EC_{90}	IC_{50}	Drug Dose	Reduction in Virus Yield	Reduction in Viral RNA
Lo 2017[39]	Remdesivir (GS5734)	Nucleoside analogue (viral replication)	Reporter assays	Hela & HEK293T/17	N/A	rNiV-M-Rluc	0.045μM	0.126μM	ND		ND	ND
						rNiV-M-ZsG	0.029μM	0.053μM	ND		ND	ND
			Virus titre reduction	Hela	N/A	NiV-B 2004	0.032μM	0.106μM	ND		ND	ND
						NiV-M 1999	0.047μM	0.083μM	ND		ND	ND
						HeV 1996	0.055μM	0.117μM	ND		ND	ND
			CPE reduction assays	Hela	N/A	NiV-M 1999	0.0655 ± 0.016μM	ND	ND	0.1μM	100%	ND
				Hela & NCI-H358		NiV-B 2004	0.0324 ± 0.0027μM	ND	ND		90%	ND
				Hela		HeV 1996	0.0548 ± 0.0013μM	ND	ND		90%	ND
			Minigenome assay	Hela	N/A	NiV-M	0.049μM	ND	ND	10μM	100%	ND
Lo 2021[40]	Remdesivir (ODBG-P-RVn)	Nucleoside analogue (viral replication)	CPE reduction assays	Vero E6	N/A	rNiV-M-ZsG	0.19 ± 0.01μM	0.30 ± 0.04μM	ND	0.8μM	100%	ND
						NiV-B	0.17 ± 0.01μM	0.38 ± 0.04μM	ND	0.8μM	100%	ND
						HeV	0.37 ± 0.04μM	3.93 ± 1.98μM	ND	0.8μM	75%	ND
				NCI-H358	N/A	NiV-B	0.82 ± 0.053μM	1.38 ± 0.05μM	ND	3μM	100%	ND
						HeV	0.95 ± 0.12μM	1.42 ± 0.03μM	ND	3μM	100%	ND
				HSAEC1-KT	N/A	rNiV-M-ZsG	0.90 ± 0.07μM	10.22 ± 4.99μM	ND	8μM	80%	ND
						NiV-B	0.41 ± 0.039μM	1.71 ± 0.66μM	ND	3μM	90%	ND
						HeV	0.42 ± 0.023μM	1.19 ± 0.061μM	ND	3μM	90%	ND
			Reporter assays	Vero E6	N/A	rNiV-M-ZsG	0.31 ± 0.04μM	0.78 ± 0.28μM	ND	0-10μM	100%	ND
				NCI-H358	N/A	rNiV-M-ZsG	0.50 ± 0.06μM	2.83 ± 1.39μM	ND	8μM	100%	ND
				HSAEC1-KT	N/A	rNiV-M-ZsG	0.57 ± 0.013μM	0.97 ± 0.21μM	ND	3μM	100%	ND
				TIME	N/A	rNiV-M-ZsG	0.75 ± 0.05μM	2.01 ± 0.30μM	ND	8μM	100%	ND
			Virus titre reduction	HSAEC1-KT	N/A	rNiV-M-ZsG	0.47μM	0.77μM	ND	20μM	3 log	ND
Dawes 2018[30]	Favipiravir (T705; 6-fluor-3-hydroxy-2-pyrazinecarbox amine)	Nucleoside analogue (viral replication)	Virus yield reduction assays	Vero	N/A	NiV-M	44.24μM	123.8μM	ND	100μM	100%	ND
						NiV-B	14.82μM	15.87μM	ND		100%	ND
						rNiV-Gluc-eGFP	14.57μM	16.25μM	ND		100%	ND
						HeV	11.71μM	16.49μM	ND		100%	ND
			Delayed treatment assay	Vero	N/A	rNiV-Gluc-eGFP	ND	ND	ND	250μM	10 fold	ND
Wright 2005[41]	Ribavirin	Nucleoside analogue (viral replication)	Virus yield reduction assays	Vero	N/A	HeV	ND	ND	ND	50μM	58 fold	9 fold

Georges-Courbot 2006[31]	Ribavirin	Nucleoside analogue (viral replication)	CPE reduction assays	Vero	Ribavirin	NiV-M	ND	ND	ND	100µg/ml 409µM	100%	ND
					EICAR	NiV-M	ND	ND	ND	1µg/ml 4.09µM	100%	ND
	6-azauridine				N/A	NiV-M	ND	ND	ND	0.25µg/ml 1.02µM	100%	ND
	Pyrazofurin				N/A	NiV-M	ND	ND	ND	0.125µg/ml 0.48µM	100%	ND
	Rintatolimod (poly I:C12U)	TLR3 agonist (host response)		Hela	N/A	NiV-M	ND	ND	ND	6.25µg/ml 6.28µM	100%	ND
Freiberg 2010[32]	Ribavirin	Nucleoside analogue (viral replication)	Virus titre reduction (dose response)	Hela	N/A	NiV-M	ND	ND	4.18µM	100µM	ND	ND
					N/A	HeV	ND	ND	4.96µM		100%	ND
	Chloroquine	Quinoline (lysosome alkalinisation)	Virus titre reduction (dose response)	Hela	N/A	NiV-M	ND	ND	0.62µM	20µM	ND	ND
					N/A	HeV	ND	ND	0.71µM		100%	ND
Porotto 2009[42]	Chloroquine	Quinoline (lysosome alkalinisation)	Multicycle assay	HEK293T co-expressing HeV G/F and venus-YFP	N/A	HeV G/F pseudotyped VSV-deltaG–RFP	ND	ND	2µM	1µM	ND	ND
			Virus titre reduction	Vero	N/A	NiV-M	ND	ND	ND	10µM	0%	30%
						HeV	ND	ND	ND		0%	75%
Lo 2020[38]	Griffithsin (GRFT)	Lectin (virus entry)	Reporter assays		GRFT	rNiV-M-rLuc	49.6 ± 19.9nM	ND	ND	10µg/mL 400nM	100%	ND
					3mG	rNiV-M-rLuc	8.4 ± 2.0nM	ND	ND	1µg/mL 40nM	100%	ND
			CPE reduction assays	Vero	GRFT	NiV-M	55.4nM	ND	ND	6.25µg/mL 250nM	100%	ND
					3mG	NiV-M	34.8nM	ND	ND	2.5µg/mL 100nM	100%	ND
					GRFT	NiV-B	41.8nM	ND	ND	6.25µg/mL 250nM	100%	ND
					3mG	NiV-B	20.1nM	ND	ND	3.75µg/mL 150nM	100%	ND
					GRFT	HeV 1996	55.1nM	ND	ND	3.75µg/mL 150nM	100%	ND
					3mG	HeV 1996	15.8nM	ND	ND	1µg/mL 40nM	100%	ND

			Virus yield reduction assays	Vero	GRFT	rNiV-M-ZsG	138.4nM	ND	ND	100µg/mL 4µM	2 log	ND
					3mG	rNiV-M-ZsG	32.1nM	ND	ND		3 log	ND
				HT-1080 & Vero	GRFT	NiV-M	42.8nM	ND	ND		4 log	ND
				Vero	GRFT	NiV-B	116.5nM	ND	ND		2 log	ND
					3mG	NiV-B	30.6nM	ND	ND		3 log	ND
Mathieu 2015[34]	Heparin	Glycosamino-glycan (virus attachment)	Inhibition of *trans*-infection	Peripheral blood leukocytes & Vero	N/A	NiV	ND	ND	ND	0-0.5mg/mL 0-33.3nM	80%	ND
				CHO-K1 & Vero	N/A	NiV	ND	ND	ND		90%	99%
			Inhibition of infection	Vero	N/A	NiV	ND	ND	ND	0.5mg/mL 33.3nM	70%	ND
						HeV	ND	ND	ND		60%	ND
Mathieu 2018[20]	Fusion inhibitory lipopeptides	Lipopeptide (virus entry)	Inhibition of cell-to-cell fusion	HEK293T	VIKI-dPEG4-Chol	N/A	ND	ND	1nM	0-10µM	ND	ND
					VIKI-dPEG4-Toco	N/A	ND	ND	7nM		ND	ND
Porotto 2010[37]					VIKI-PEG4-Chol	NiV G/F protein co-expressing cells	ND	ND	5nM	1µM	ND	ND
Welch 2020[43]	Defective interfering particles	Virus-like particles (viral replication)	Virus yield reduction assays	Vero	DI-01	rNiV-M/ZsG	ND	ND	ND	5000:1 DIP to NiV genome ratio	100 fold	ND
						NiV-M	ND	ND	ND		90 fold	ND
						NiV-B	ND	ND	ND		30 fold	ND
					DI-03	rNiV-M/ZsG	ND	ND	ND		100 fold	ND
						NiV-M	ND	ND	ND		90 fold	ND
						NiV-B	ND	ND	ND		20 fold	ND
					DI-07	rNiV-M/ZsG	ND	ND	ND		900 fold	ND
						NiV-M	ND	ND	ND		500 fold	ND
						NiV-B	ND	ND	ND		80 fold	ND
					DI-10	rNiV-M/ZsG	ND	ND	ND		1000 fold	ND
						NiV-M	ND	ND	ND		700 fold	ND
						NiV-B	ND	ND	ND		500 fold	ND
					DI-14	rNiV-M/ZsG	ND	ND	ND		1000 fold	ND
						NiV-M	ND	ND	ND		1000 fold	ND
						NiV-B	ND	ND	ND		600 fold	ND
					DI-15	rNiV-M/ZsG	ND	ND	ND		1000 fold	ND
						NiV-M	ND	ND	ND		800 fold	ND
						NiV-B	ND	ND	ND		100 fold	ND
					DI-16	rNiV-M/ZsG	ND	ND	ND		900 fold	ND
						NiV-M	ND	ND	ND		800 fold	ND
						NiV-B	ND	ND	ND		90 fold	ND

					DI-35	rNiV-M/ZsG	ND	ND	ND		1000 fold	ND
						NiV-M	ND	ND	ND		900 fold	ND
						NiV-B	ND	ND	ND		400 fold	ND
					DI-dTom	rNiV-M/ZsG	ND	ND	ND		80 fold	ND
						NiV-M	ND	ND	ND		80 fold	ND
						NiV-B	ND	ND	ND		80 fold	ND

CHO = Chinese hamster ovary; CPE = cytopathic effect; DIP = defective interfering particles; EC_{50} = 50% maximal effective concentration; EC_{90} = 90% maximal effective concentration; eGFP = enhanced Green Fluorescent Protein; EICAR = 5-Ethynyl-1-beta-D-ribofuranosyllmidazole-4-CARboxamide; Gluc = *Gaussia* luciferase; GRFT = griffithsin; HEK = human embryonic kidney; HeV = Hendra virus; HSAEC1 = human small airway epithelial cells; hTERT = human telomerase reverse transcriptase; IC_{50} = 50% maximal inhibitory concentration; N/A = not applicable; NCI = National Cancer Institute; ND = not done; NiV-B = Nipah virus Bangladesh; NiV-M = Nipah virus Malaysia; Rluc = *Renilla* luciferase; rNIV = recombinant Nipah virus; RFP = red fluorescent protein; TIME = hTERT immortalised microvascular endothelial cells; TLR3 = toll-like receptor 3; 3mG = trimeric monomeric griffithsin; VSV = vesicular stomatitis virus; YFP = yellow fluorescent protein; ZsG = *Zoanthus* sp. green fluorescent protein.

Table IV: Nipah & Hendra Virus Therapeutic Small Molecules (Exploratory *In Vitro* Studies)

Reference	Small Molecule (Mechanism)	Efficacy (Assay)	Safety (Assay)	Suitability for Animal Studies
Aljofan 2010[44]	Calcium flux modulators (viral replication inhibitors): 41 repurposed compounds	IC_{50} values:<br>Micromolar to millimolar concentrations<br>(High throughput screening immunolabelling assay with NiV-M and HeV on Vero cells)	CC_{50} values:<br>Micromolar to millimolar concentrations<br>(CellTiter-Glo assay on Vero cells)	Potentially. A number are known toxins, while others are licensed drugs in widespread use.
Aljofan 2009[45]	Brilliant green, gentian violet, gliotoxin (mechanism unknown)	IC_{50} values:<br>Brilliant green = 218nM (NiV-M), 778nM (HeV)<br>Gentian violet = 525nM (NiV-M), 2679nM (HeV)<br>Gliotoxin = 149nM (NiV-M), 579nM (HeV)<br>(High throughput screening immunolabelling assay with NiV-M and HeV on Vero cells)	CC_{50} values:<br>Brilliant green = 4672nM (293T), 861nM (Vero)<br>Gentian violet = 5865nM (293T), 2828nM (Vero)<br>Gliotoxin = 4896nM (293T), 1609nM (Vero)<br>(CellTIter-Glo assay in 293T cells and alamarBlue in Vero cells)	No. Dyes too toxic for systemic use which could instead be considered for topical use or decontamination of surfaces.
Elshabrawy 2014[46]	Cathepsin L inhibitors (viral entry inhibitors): 5705213, 7402683	EC_{50}/IC_{50} values not given<br>5705213 and 7402683 inhibited NiV and HeV pseudovirus entry by ~80% and ~90% at 100µM respectively<br>(Viral entry assays with NiV and HeV pseudoviruses on 293FT cells)	CC_{50} values:<br>5705213 = 400µM<br>7402683 = 350µM<br>(MTT assay in 293FT cells)	Potentially. Novel compounds identified through a high-throughput screening assay. Need further testing with live viruses.
Hotard 2017[47]	R1479 (nucleoside analogue)	EC_{50} values (varying by assay and cell line):<br>R1479 = 1.53-13.55µM<br>(Reporter rNiV assays, CPE and titre reduction assays using NiV-M and HeV, all in NCI-H358 and HeLa cells)	CC_{50} value:<br>R1479 >100µM<br>(CellTiter-Glo assay in NCI-H358 cells)	No. R1479 is metabolite of balapiravir which is inhibited by cytokines produced in dengue infection[48] and is associated with dose-dependent lymphopenia.
Janardhana 2012[49]	Recombinant bat interferon-gamma (host immunomodulator)	EC_{50}/IC_{50} values not given<br>Bat IFN-γ significantly reduced number of HeV positive cells<br>(Immunolabelling assay in bat kidney cells using HeV)	Not tested	Potentially. First evidence of antiviral role of bat IFN-γ.
Liu 2013[50]	25-hydroxycholesterol (viral replication and fusion inhibitor)	EC_{50}/IC_{50} values not given<br>25HC 5µM reduced viral titres by ~2 log at 72HPI<br>25HC 2µM reduced fusion by ~50% and 10µM by ~60%<br>(Titre reduction assay using NIV-B in HeLa cells & fusion assay using rNiV in Vero cells)	Lactate dehydrogenase level increased only after 30-40h of treatment at 40µM of 25HC<br>(Adenosine triphosphate and lactate dehydrogenase assays on HEK293 cells)	Potentially. Reduced HIV infection in humanised mice in separate experiment in same publication.
Lo 2020[33]	ALS-8112 (nucleoside analogue)	EC_{50} values (varying by assay and cell line):<br>ALS-8112 = 0.30-3.08µM<br>(Reporter rNiV assays, CPE and titre reduction assays using NiV-M and NiV-B, all in NCI-H358 and HSAEC1-KT cells)	CC_{50} values:<br>ALS-8112 >50µM<br>(CellTiterGlo assay in NCI-H358 and HSAEC1-KT cells)	Potentially. ALS-8112 is parent drug of lumicitabine which was withdrawn from development for RSV due to paediatric neutropenia[51].
Lo 2018[52]	R1479 with 2'-monofluoro- or 2'-difluoro-modifications (nucleoside analogues)	EC_{50} values (varying by assay and cell line):<br>R1479 = 1.5-3.1µM<br>2'-monofluoro-R1479 = 0.14-0.37µM<br>2'-difluoro-R1479 = 0.15-0.57µM<br>(Reporter rNIV assays, CPE and titre reduction assays using NiV-M and HeV, all in NCI-H358 and HeLa cells)	CC_{50} values:<br>R1479 >100µM<br>2'-monofluoro-R1479 >100µM<br>2'-difluoro-R1479 >100µM<br>(CellTiter-Glo assay on NCI-H358 cells)	No. Greater potency with 2'-fluoro-modified analogues than R1479 but variable incorporation of all by host mitochondrial RNA and DNA polymerases limits viability.

McCaskill 2013[53]	Polyinosinic:polycytidylic acid (TLR3 agonist) + small interfering ribonucleic acids (RNA interference)	EC_{50}/IC_{50} values not given<br>Poly I:C 1µg/ml + siRNA 1nM induced >98% (~1.5 log) reduction in HeV titre<br>(Titre reduction assay using HeV on HeLa cells)	Not tested	No. Poly I:C toxicity along siRNA specificity, stability, and delivery challenges are limiting factors.
Mohr 2015[54]	OSU-03012 (host cell kinase inhibitor)	EC_{50} value:<br>OSU-03012 = 0.4µM<br>(Reporter assay using rNiV-luciferase on HEK293 cells)	CC_{50} value:<br>OSU-03012 = 8.2µM<br>(CellTiter-Glo assay on HEK293 cells)	Potentially. Celecoxib oral derivative discontinued from development for poor absorption and bioavailability.
Mungall 2008[55]	Small interfering ribonucleic acids (RNA interference)	EC_{50}/IC_{50} values not given<br>Three siRNAs each at 50nM reduced replication by >60%<br>(Immunolabelling assay of NiV-M on BHK-21 cells)	Not tested	No. Challenges with specificity, stability, and delivery.
Niedemeier 2009[56]	Hydroxyquinoline compounds (viral fusion inhibitors): compound 19	EC_{50} value:<br>Compound 19 = 1.5µM<br>(Cell fusion assay with NiV-M in Vero cells)	CC_{50} value:<br>Compound 19 >20µM<br>(MTT assay on Vero cells)	Potentially. Small molecule inhibitor identified through *in silico* screen. Compound 19 most active inhibitor of nine.
Pattabhi 2016[57]	Hydroxyquinoline compounds (interferon regulatory factor 3 activation): KIN1408	EC_{50}/IC_{50} values not given<br>KIN1408 5µM caused 1.5 log unit decrease in infectious NiV<br>(Treated HUVECs infected with NiV-M then cell culture supernatant analysed by plaque assay on Vero cells)	CC_{50} value:<br>KIN1408 >50µM<br>(CellTiter 96 Aqueous cell proliferation assay with HEK293 or HuH7 cells)	Potentially. Derivative of hydroxyquinoline compound KIN1400 identified from a cell-based screen.
Porotto 2011[58]	Synthetic protocells (viral fusion inhibitors)	EC_{50}/IC_{50} values not given<br>(Infection assay using NiV and HeV pseudovirus)	Not tested	No. Artificial cell-like particles. Unclear if sufficiently stable for *in vivo* testing.
Pu 2022[59]	Furanyl methylidene rhodanine analogues (viral fusion inhibitors): FD001, FD012	IC_{50} values:<br>FD001 = 0.41±0.07µM<br>FD0012 = 0.07±0.01µM<br>(Inhibition assay using NiV pseudovirus in U87 cells)	CC_{50} values:<br>FD001 >50µM<br>FD0012 41.69µM<br>(Cell Counting Kit-8 on U87 cells)	Potentially. Synthesised novel compounds. Need further testing with live virus.
Shrestha 2021[60]	Saturated fused thiazole derivative compounds (viral replication inhibitors): ZHAWOC21026	IC_{50} value:<br>ZHAWOC21026 = 0.08µM<br>(Reporter assay using rNiV-eGFP in CHO pgsA-745 cells transfected with human ephrin-B2)	CC_{50} value:<br>ZHAWOC21026 = 80µM<br>(RealTime-Glo MT assay on CHO pgsA-745 cells transfected with human ephrin-B2)	Potentially. Optimised novel compound identified through a high-throughput screening assay.
Tigabu 2014[61]	Sulfonamide compounds (mechanism unknown): AB00991123, AB00992391, AB003210	EC_{50} values:<br>AB00991123 = 3.9µM<br>AB00992391 = 11.7µM<br>AB003210 = 7.8µM<br>(Titre reduction assays using NiV-M on Vero cells)	CC_{50}/EC_{50} selectivity indices:<br>AB00991123 >40<br>AB00992391 >12<br>AB003210 >18<br>(Viral ToxGlo assay on Vero cells)	Potentially. Novel compounds identified through a high-throughput screening assay.
Wang 2010[62]	Bortezomib & MG132<br>(host cell proteasome inhibitors)	IC_{50} values:<br>Bortezomib = 2.7nM<br>MG132 = 0.47nM<br>(Dose-response inhibition assays using NiV-M on HeLa cells)	CC_{50} values:<br>Bortezomib >2.5µM<br>MG132 >2.5µM<br>(ToxiLight BioAssay kit on HeLa cells)	Potentially. Bortezomib is USA FDA-approved for mantle cell lymphoma. MG132 has limited *in vivo* utility due to configurational instability.
Wolf 2010[63]	LJ001 (viral entry inhibitor)	IC_{50} values:<br>LJ001 = 0.5-1µM	*In vitro*:<br>Not toxic at effective antiviral concentrations.	No. Poor physiological stability. Requires light for antiviral

		(Titre reduction assay using NiV-M on Vero cells)	(Adenylate kinase, lactate dehydrogenase, and alamarBlue assays on Vero cells)<br>*In vivo:*<br>No toxicity observed in female BALB/c mice dosed PO or IP with 20mg/kg or 50mg/kg of compound, other than slight elevation of serum cholesterol levels.	mechanism.

25HC = 25-hydroxycholesterol; BHK = baby hamster kidney; CC_{50} = 50% cytotoxicity concentration; CHO = Chinese Hamster Ovary; CPE = cytopathic effect; DNA = deoxyribonucleic acid; EC_{50} = 50% maximal effective concentration; eGFP = enhanced Green Fluorescent Protein; FDA = Food and Drug Administration; GFP = green fluorescent protein; HEK = human embryonic kidney; HeV = Hendra virus; HIV = human immunodeficiency virus; HPI = hours post infection; HSAEC = human small airway epithelial cells; HuH = human hepatoma; HUVEC = human umbilical vein endothelial cell; IC_{50} = 50% maximal inhibitory concentration; IFN-γ = interferon gamma; IP = intraperitoneal; MTT = 3-(4,5-dimethylthiazol-2-yl)-2,5-diphenyltetrazolium bromide; NCI = National Cancer Institute; NiV = Nipah virus; NiV-B = Nipah virus Bangladesh; NiV-M = Nipah virus Malaysia; PFU = plaque forming units; PO = orally (per os); RNA = ribonucleic acid; rNiV = recombinant Nipah virus; RSV = respiratory syncytial virus; siRNA = small interfering ribonucleic acid; TLR3 = toll-like receptor 3; USA = United States of America.

Table V: Nipah & Hendra Virus Therapeutics Animal Challenge Studies by Drug, Viral Challenge Strain, and Animal Model

**Drug**	**Nipah Virus Malaysia (NiV-M)**	**Nipah Virus Bangladesh (NiV-B)**	**Hendra Virus (HeV)**
Monoclonal Antibodies			
m102.4	African green monkeys[13]<br>• 5×10^5 PFU intratracheal	African green monkeys[12]<br>• 2.5×10^5 PFU intratracheal + 2.5×10^5 PFU intranasal	African green monkeys[14]<br>• 4×10^5 $TCID_{50}$ intratracheal
	Ferrets[23]<br>• 5×10^3 $TCID_{50}$ oronasal		
1F5 vs m102.4		African green monkeys[15]<br>• 4×10^4 PFU intranasal	
1F5 vs 12B2 vs 1F5 + 12B2		Syrian golden hamsters[15]<br>• 5×10^6 PFU intranasal	
h5B3.1	Ferrets[22]<br>• 5×10^3 PFU intranasal		Ferrets[22]<br>• 5×10^3 PFU intranasal
NiV41		Syrian golden hamsters[26]<br>• 10^5 $TCID_{50}$ intraperitoneal	
NiV41-6	Syrian golden hamsters[26]<br>• 1000 LD_{50} intraperitoneal		
HENV-103, HENV-117, HENV-58, HENV-98, HENV-100		Syrian golden hamsters[27]<br>• 5×10^6 PFU intranasal	
HENV-26, HENV-32		Ferrets[21]<br>• 5×10^3 PFU intranasal	
NipGIP1.7, Nip3B10, NipGIP35, NipGIP3	Syrian golden hamsters[28]<br>• 7.5×10^2 PFU (100 LD_{50}) intraperitoneal		
NipGIP35, NipGIP3, NipGIP21, NipGIP7			Syrian golden hamsters[29]<br>• 10^3 PFU (100 LD_{50}) intraperitoneal
Small Molecules			
Remdesivir		African green monkeys[16-18]<br>• 10^5 $TCID_{50}$ intratracheal + 10^5 $TCID_{50}$ intranasal	
Favipiravir	Syrian golden hamsters[30]<br>• 10^4 PFU intraperitoneal		

Ribavirin			African green monkeys[19]<br>• 4 x 10^5 $TCID_{50}$ intratracheal
Ribavirin vs 6-azauridine vs Rintatolimod	Syrian golden hamsters[31]<br>• Experiment 1: 350 LD_{50} intraperitoneal<br>• Experiment 2: 35 LD_{50} intraperitoneal		
Ribavirin vs Chloroquine vs Ribavirin + Chloroquine	Syrian golden hamsters[32]<br>• 10^4 $TCID_{50}$ intraperitoneal		Syrian golden hamsters[32]<br>• 10^4 $TCID_{50}$ intraperitoneal
Chloroquine	Ferrets[25]<br>• 5 x 10^3 $TCID_{50}$ (10 LD_{50}) oronasal		
Griffithsin		Syrian golden hamsters[38]<br>• 10^7 $TCID_{50}$ intranasal	
Periodate heparin	Syrian golden hamsters[34]<br>• 500 LD_{50} intraperitoneal		
Fusion inhibitory lipopeptides	African green monkeys[20]<br>• 2 x 10^7 PFU intratracheal		
	Syrian golden hamsters[20,35]<br>• 10^6 PFU (100 LD_{50}) intranasal[20]<br>• 100 LD_{50} intraperitoneal[35]		
Defective interfering particles	Syrian golden hamsters[36]<br>• 10^4 $TCID_{50}$ intraperitoneal or 10^6 $TCID_{50}$ intranasal		

LD_{50} = median lethal dose, PFU = plaque forming units, $TCID_{50}$ = median tissue culture infectious dose.

One study of lipopeptides in Syrian golden hamsters[37] did not specify the Nipah virus strain used.

Risk of Bias Assessments – Additional Figures

Figure I: Risk of Bias Assessment of Randomised Clinical Trials by Individual Study

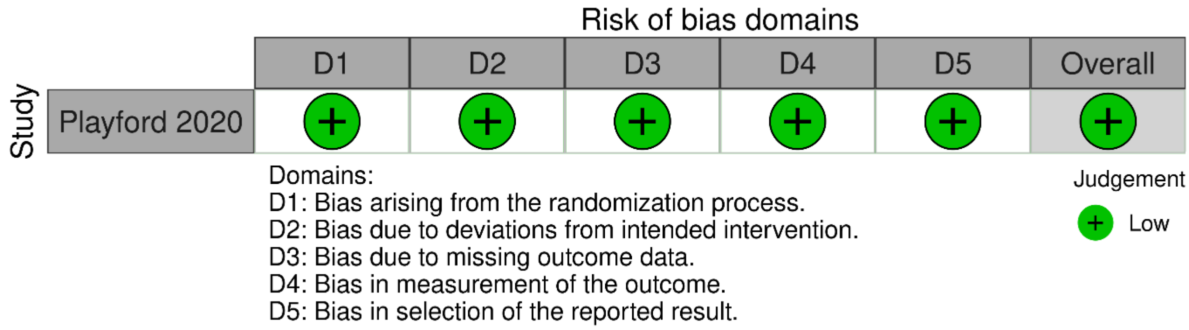

Figure II: Summary Risk of Bias Assessment of Non-randomised Clinical Studies

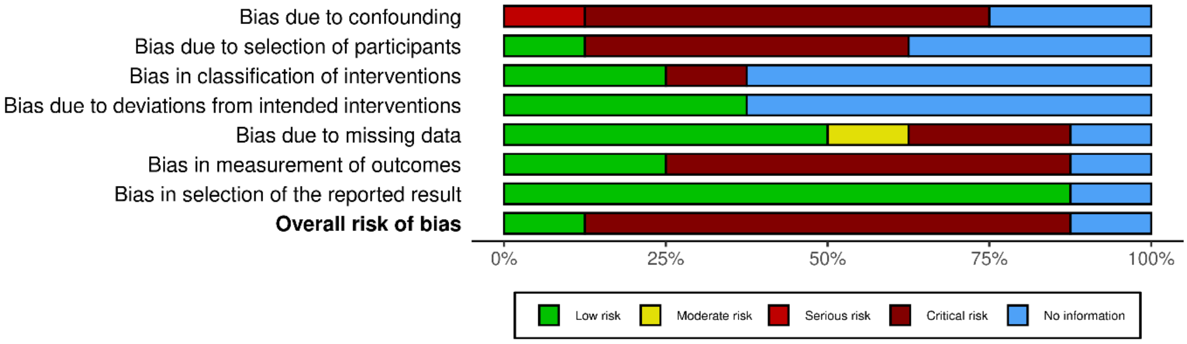

Figure III: Risk of Bias Assessment of Observational Studies by Individual Study

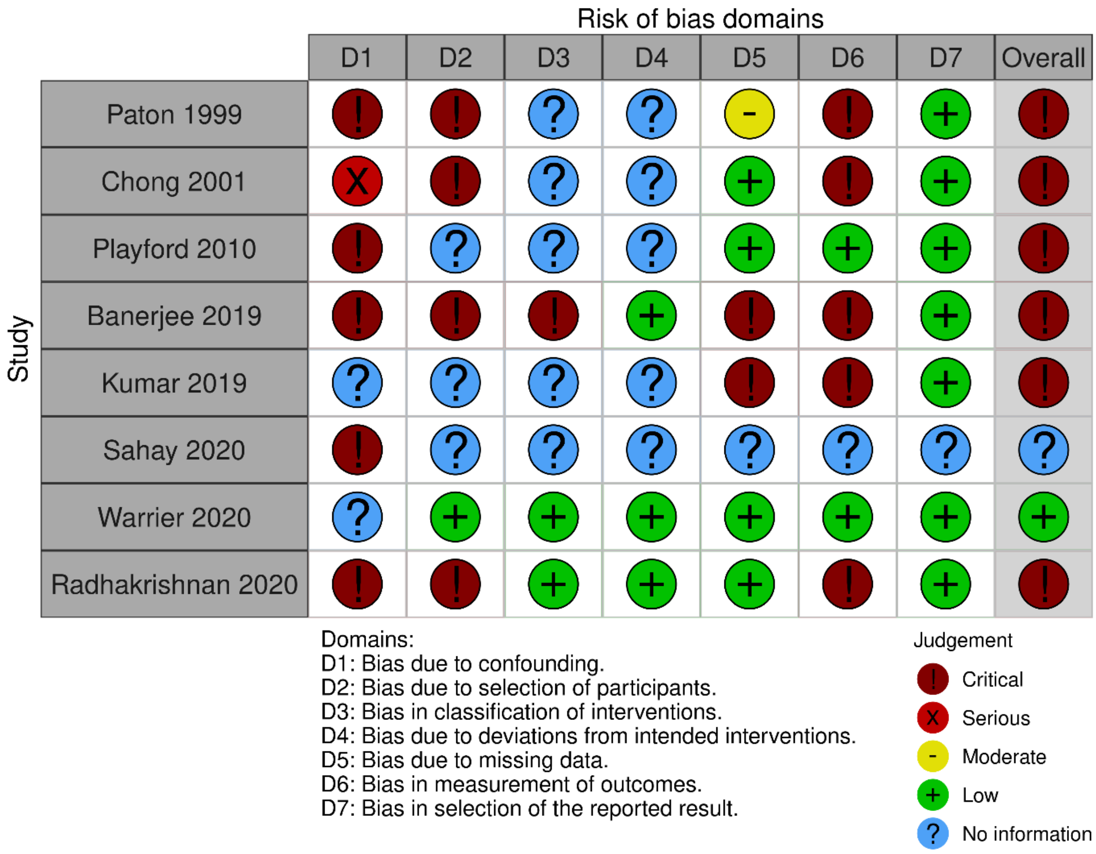

Figure IV: Summary Risk of Bias Assessment of Animal Studies

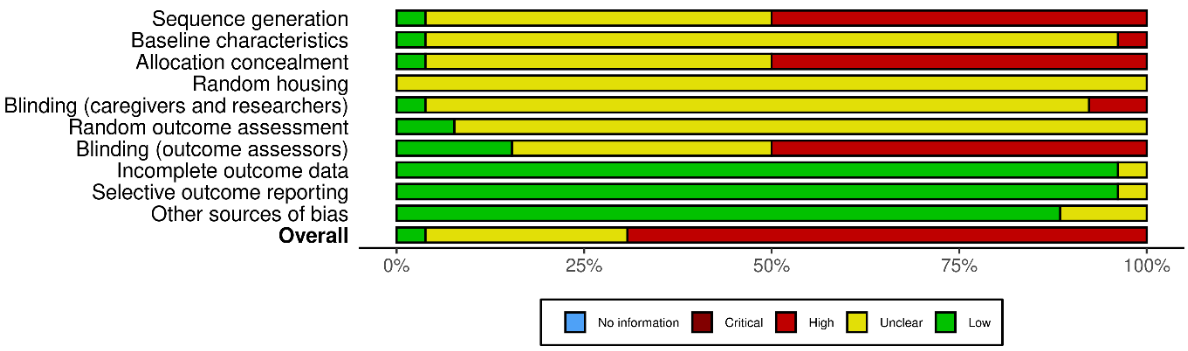

Figure V: Risk of Bias Assessment of Animal Studies by Individual Study

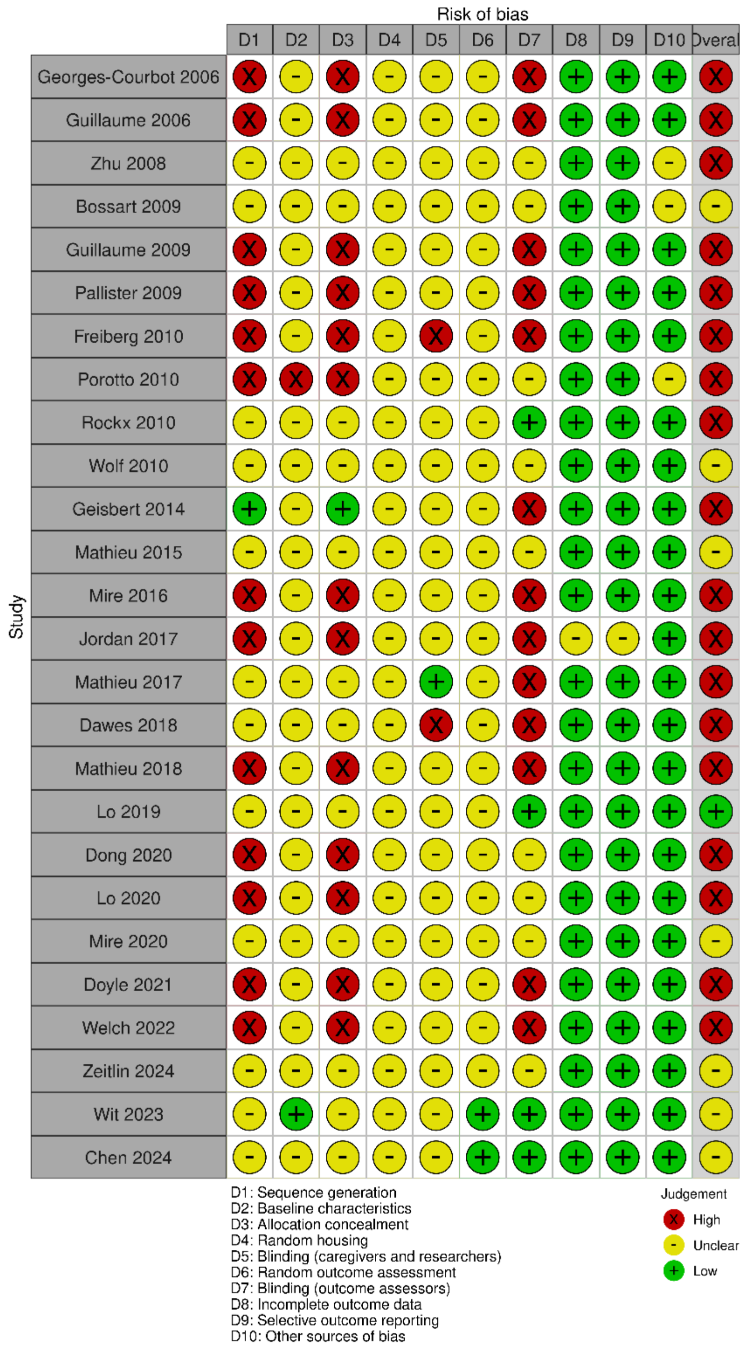

PRISMA 2020 CHECKLIST

Section and Topic	Item #	Checklist item	Location where item is reported
**TITLE**			
Title	1	Identify the report as a systematic review.	Title
**ABSTRACT**			
Abstract	2	See the PRISMA 2020 for Abstracts checklist.	Abstract
**INTRODUCTION**			
Rationale	3	Describe the rationale for the review in the context of existing knowledge.	Introduction
Objectives	4	Provide an explicit statement of the objective(s) or question(s) the review addresses.	Introduction
**METHODS**			
Eligibility criteria	5	Specify the inclusion and exclusion criteria for the review and how studies were grouped for the syntheses.	Methods – Eligibility Criteria<br>Methods – Data Analysis
Information sources	6	Specify all databases, registers, websites, organisations, reference lists and other sources searched or consulted to identify studies. Specify the date when each source was last searched or consulted.	Methods – Search Strategy
Search strategy	7	Present the full search strategies for all databases, registers and websites, including any filters and limits used.	Supplementary Methods – Search Strategies
Selection process	8	Specify the methods used to decide whether a study met the inclusion criteria of the review, including how many reviewers screened each record and each report retrieved, whether they worked independently, and if applicable, details of automation tools used in the process.	Methods – Review Team & Tools
Data collection process	9	Specify the methods used to collect data from reports, including how many reviewers collected data from each report, whether they worked independently, any processes for obtaining or confirming data from study investigators, and if applicable, details of automation tools used in the process.	Methods – Data Extraction<br>Methods – Review Team & Tools
Data items	10a	List and define all outcomes for which data were sought. Specify whether all results that were compatible with each outcome domain in each study were sought (e.g. for all measures, time points, analyses), and if not, the methods used to decide which results to collect.	Methods – Data Extraction
	10b	List and define all other variables for which data were sought (e.g. participant and intervention characteristics, funding sources). Describe any assumptions made about any missing or unclear information.	Methods – Data Extraction
Study risk of bias assessment	11	Specify the methods used to assess risk of bias in the included studies, including details of the tool(s) used, how many reviewers assessed each study and whether they worked independently, and if applicable, details of automation tools used in the process.	Methods – Quality Assessment<br>Methods – Review Team & Tools

Section and Topic	Item #	Checklist item	Location where item is reported
Effect measures	12	Specify for each outcome the effect measure(s) (e.g. risk ratio, mean difference) used in the synthesis or presentation of results.	Methods – Data Analysis
Synthesis methods	13a	Describe the processes used to decide which studies were eligible for each synthesis (e.g. tabulating the study intervention characteristics and comparing against the planned groups for each synthesis (item #5)).	Methods – Data Analysis
	13b	Describe any methods required to prepare the data for presentation or synthesis, such as handling of missing summary statistics, or data conversions.	N/A
	13c	Describe any methods used to tabulate or visually display results of individual studies and syntheses.	Methods – Data Analysis
	13d	Describe any methods used to synthesize results and provide a rationale for the choice(s). If meta-analysis was performed, describe the model(s), method(s) to identify the presence and extent of statistical heterogeneity, and software package(s) used.	Methods – Data Analysis
	13e	Describe any methods used to explore possible causes of heterogeneity among study results (e.g. subgroup analysis, meta-regression).	N/A
	13f	Describe any sensitivity analyses conducted to assess robustness of the synthesized results.	N/A
Reporting bias assessment	14	Describe any methods used to assess risk of bias due to missing results in a synthesis (arising from reporting biases).	Methods – Quality Assessment
Certainty assessment	15	Describe any methods used to assess certainty (or confidence) in the body of evidence for an outcome.	N/A
**RESULTS**			
Study selection	16a	Describe the results of the search and selection process, from the number of records identified in the search to the number of studies included in the review, ideally using a flow diagram.	Figure 1
	16b	Cite studies that might appear to meet the inclusion criteria, but which were excluded, and explain why they were excluded.	Results – Included Studies<br>Supplementary Results – Included Studies
Study characteristics	17	Cite each included study and present its characteristics.	Tables 1-2, Supplementary Tables I-V
Risk of bias in studies	18	Present assessments of risk of bias for each included study.	Supplementary Figures I-V
Results of individual studies	19	For all outcomes, present, for each study: (a) summary statistics for each group (where appropriate) and (b) an effect estimate and its precision (e.g. confidence/credible interval), ideally using structured tables or plots.	N/A

Section and Topic	Item #	Checklist item	Location where item is reported
Results of syntheses	20a	For each synthesis, briefly summarise the characteristics and risk of bias among contributing studies.	Results – Risk of Bias
	20b	Present results of all statistical syntheses conducted. If meta-analysis was done, present for each the summary estimate and its precision (e.g. confidence/credible interval) and measures of statistical heterogeneity. If comparing groups, describe the direction of the effect.	N/A
	20c	Present results of all investigations of possible causes of heterogeneity among study results.	N/A
	20d	Present results of all sensitivity analyses conducted to assess the robustness of the synthesized results.	N/A
Reporting biases	21	Present assessments of risk of bias due to missing results (arising from reporting biases) for each synthesis assessed.	N/A
Certainty of evidence	22	Present assessments of certainty (or confidence) in the body of evidence for each outcome assessed.	N/A
**DISCUSSION**			
Discussion	23a	Provide a general interpretation of the results in the context of other evidence.	Discussion
	23b	Discuss any limitations of the evidence included in the review.	Discussion
	23c	Discuss any limitations of the review processes used.	Discussion
	23d	Discuss implications of the results for practice, policy, and future research.	Discussion
**OTHER INFORMATION**			
Registration and protocol	24a	Provide registration information for the review, including register name and registration number, or state that the review was not registered.	Methods
	24b	Indicate where the review protocol can be accessed, or state that a protocol was not prepared.	Methods
	24c	Describe and explain any amendments to information provided at registration or in the protocol.	N/A
Support	25	Describe sources of financial or non-financial support for the review, and the role of the funders or sponsors in the review.	Methods – Role of the Funding Source<br>Acknowledgements
Competing interests	26	Declare any competing interests of review authors.	N/A
Availability of data, code and other materials	27	Report which of the following are publicly available and where they can be found: template data collection forms; data extracted from included studies; data used for all analyses; analytic code; any other materials used in the review.	Methods – Data Extraction & Data Analysis

From: Page MJ, McKenzie JE, Bossuyt PM, Boutron I, Hoffmann TC, Mulrow CD, et al. The PRISMA 2020 statement: an updated guideline for reporting systematic reviews. BMJ 2021;372:n71. doi: 10.1136/bmj.n71

For more information, visit: http://www.prisma-statement.org/

References

1. Playford EG, Munro T, Mahler SM, et al. Safety, tolerability, pharmacokinetics, and immunogenicity of a human monoclonal antibody targeting the g glycoprotein of henipaviruses in healthy adults: a first-in-human, randomised, controlled, phase 1 study. *Lancet Infectious Diseases* 2020; **20**(4): 445-54.
2. Playford EG, McCall B, Smith G, et al. Human Hendra virus encephalitis associated with equine outbreak, Australia, 2008. *Emerging Infectious Diseases* 2010; **16(2)**: 219-23.
3. Banerjee S, Niyas VKM, Soneja M, et al. First experience of ribavirin postexposure prophylaxis for Nipah virus, tried during the 2018 outbreak in Kerala, India. *Journal of Infection* 2019; **78(6)**: 491-503.
4. Kumar ASA, Sohanlal T, Prasad G, Gupta M, Gopal A. Single-center experience and lessons learnt from management of NIPAH virus outbreak in India. *Open Forum Infectious Diseases* 2019; **6(Supplement 2)**: S659-S60.
5. Radhakrishnan C, Renjith TP, Arshad F, et al. Clinical manifestations of Nipah virus-infected patients who presented to the emergency department during an outbreak in Kerala state in India, May 2018. *Clinical Infectious Diseases* 2020; **71**(1): 152-7.
6. Warrier A. A single case outbreak of Nipah Encephalitis from India in May-June 2019. *International Journal of Infectious Diseases* 2020; **101(Supplement 1)**: 247.
7. Sahay RR, Yadav PD, Gupta N, et al. Experiential learnings from the Nipah virus outbreaks in Kerala towards containment of infectious public health emergencies in India. *Epidemiol Infect* 2020; **148**: e90.
8. Paton NI, Leo Y, Zaki SR, et al. Outbreak of Nipah-virus infection among abattoir workers in Singapore. *Lancet* 1999; **354**(9186): 1253-6.
9. Chong HT, Kamarulzaman A, Tan CT, et al. Treatment of acute Nipah encephalitis with ribavirin. *Annals of Neurology* 2001; **49(6)**: 810-3.
10. Tan CT, Wong KT. Nipah encephalitis outbreak in Malaysia. *Annals of the Academy of Medicine Singapore* 2003; **32(1)**: 112-7.
11. Gayathri K. An observational study in the setting of Nipah virus outbreak Kerala 2018. *Lung India* 2019; **36(9 Supplement)**: S172-S3.
12. Mire CE, Satterfield BA, Geisbert JB, et al. Pathogenic Differences between Nipah Virus Bangladesh and Malaysia Strains in Primates: Implications for Antibody Therapy. *Scientific Reports* 2016; **6**.
13. Geisbert TW, Mire CE, Geisbert JB, et al. Therapeutic treatment of Nipah virus infection in nonhuman primates with a neutralizing human monoclonal antibody. *Science Translational Medicine* 2014; **6(242) (no pagination)**.
14. Bossart KN, Geisbert TW, Feldmann H, et al. A Neutralizing Human Monoclonal Antibody Protects African Green Monkeys from Hendra Virus Challenge. *Science Translational Medicine* 2011; **3**(105).
15. Zeitlin L, Cross RW, Woolsey C, et al. Therapeutic administration of a cross-reactive mAb targeting the fusion glycoprotein of Nipah virus protects nonhuman primates. *Sci Transl Med* 2024; **16**(741): eadl2055.
16. de Wit E, Williamson BN, Feldmann F, et al. Late remdesivir treatment initiation partially protects African green monkeys from lethal Nipah virus infection. *Antiviral Res* 2023; **216**: 105658.
17. Lo MK, Feldmann F, Gary JM, et al. Remdesivir (GS-5734) protects African green monkeys from Nipah virus challenge. *Sci Transl Med* 2019; **11**(494).
18. Jordan R, Hogg A, Warren T, et al. Broad-spectrum investigational agent GS-5734 for the treatment of ebola, mers coronavirus and other pathogenic viral infections with high outbreak potential. *Open Forum Infectious Diseases* 2017; **4(Supplement 1)**: S737.

19. Rockx B, Bossart KN, Feldmann F, et al. A novel model of lethal Hendra virus infection in African green monkeys and the effectiveness of ribavirin treatment. *Journal of Virology* 2010; **84(19)**: 9831-9.
20. Mathieu C, Porotto M, Figueira TN, Horvat B, Moscona A. Fusion Inhibitory Lipopeptides Engineered for Prophylaxis of Nipah Virus in Primates. *J Infect Dis* 2018; **218**(2): 218-27.
21. Dong J, Cross RW, Doyle MP, et al. Potent Henipavirus Neutralization by Antibodies Recognizing Diverse Sites on Hendra and Nipah Virus Receptor Binding Protein. *Cell* 2020; **183**(6): 1536-50.e17.
22. Mire CE, Chan Y-P, Borisevich V, et al. A Cross-Reactive Humanized Monoclonal Antibody Targeting Fusion Glycoprotein Function Protects Ferrets Against Lethal Nipah Virus and Hendra Virus Infection. *Journal of Infectious Diseases* 2020; **221**: S471-S9.
23. Bossart KN, Zhu Z, Middleton D, et al. A neutralizing human monoclonal antibody protects against lethal disease in a new ferret model of acute Nipah virus infection. *PLoS Pathogens* 2009; **5(10) (no pagination)**.
24. Zhu Z, Bossart KN, Bishop KA, et al. Exceptionally potent cross-reactive neutralization of Nipah and Hendra viruses by a human monoclonal antibody. *Journal of Infectious Diseases* 2008; **197(6)**: 846-53.
25. Pallister J, Middleton D, Crameri G, et al. Chloroquine administration does not prevent Nipah virus infection and disease in ferrets. *Journal of Virology* 2009; **83(22)**: 11979-82.
26. Chen L, Sun M, Zhang H, et al. Potent human neutralizing antibodies against Nipah virus derived from two ancestral antibody heavy chains. *Nat Commun* 2024; **15**(1): 2987.
27. Doyle MP, Kose N, Borisevich V, et al. Cooperativity mediated by rationally selected combinations of human monoclonal antibodies targeting the henipavirus receptor binding protein. *Cell Reports* 2021; **36(9) (no pagination)**.
28. Guillaume V, Contamin H, Loth P, et al. Antibody prophylaxis and therapy against Nipah virus infection in hamsters. *Journal of Virology* 2006; **80(4)**: 1972-8.
29. Guillaume V, Wong KT, Looi RY, et al. Acute Hendra virus infection: Analysis of the pathogenesis and passive antibody protection in the hamster model. *Virology* 2009; **387**(2): 459-65.
30. Dawes BE, Kalveram B, Ikegami T, et al. Favipiravir (T-705) protects against Nipah virus infection in the hamster model. *Scientific Reports* 2018; **8**(1).
31. Georges-Courbot MC, Contamin H, Faure C, et al. Poly(I)-poly(C12U) but not ribavirin prevents death in a hamster model of Nipah virus infection. *Antimicrob Agents Chemother* 2006; **50**(5): 1768-72.
32. Freiberg AN, Worthy MN, Lee B, Holbrook MR. Combined chloroquine and ribavirin treatment does not prevent death in a hamster model of Nipah and Hendra virus infection. *Journal of General Virology* 2010; **91(3)**: 765-72.
33. Lo MK, Amblard F, Flint M, et al. Potent in vitro activity of β-D-4′-chloromethyl-2′-deoxy-2′-fluorocytidine against Nipah virus. *Antiviral Research* 2020; **175**.
34. Mathieu C, Dhondt KP, Chalons M, et al. Heparan sulfate-dependent enhancement of henipavirus infection. *Mbio* 2015; **6(2) (no pagination)**.
35. Mathieu C, Augusto MT, Niewiesk S, et al. Broad spectrum antiviral activity for paramyxoviruses is modulated by biophysical properties of fusion inhibitory peptides. *Scientific Reports* 2017; **7**.
36. Welch SR, Spengler JR, Harmon JR, et al. Defective Interfering Viral Particle Treatment Reduces Clinical Signs and Protects Hamsters from Lethal Nipah Virus Disease. *Mbio* 2022; **13**(2): e0329421.
37. Porotto M, Rockx B, Yokoyama CC, et al. Inhibition of Nipah Virus Infection In Vivo: Targeting an early stage of paramyxovirus fusion activation during viral entry. *PLoS Pathogens* 2010; **6(10) (no pagination)**.

38. Lo MK, Spengler JR, Krumpe LRH, et al. Griffithsin inhibits Nipah virus entry and fusion and can protect Syrian golden hamsters from lethal Nipah virus challenge. (Special Issue: Twenty years of Nipah virus research.). *Journal of Infectious Diseases* 2020; **221**(Suppl. 4): S480-S92.
39. Lo MK, Jordan R, Arvey A, et al. GS-5734 and its parent nucleoside analog inhibit Filo-, Pneumo-, and Paramyxoviruses. *Scientific Reports* 2017; **7**.
40. Lo MK, Shrivastava-Ranjan P, Chatterjee P, et al. Broad-Spectrum In Vitro Antiviral Activity of ODBG-P-RVn: An Orally-Available, Lipid-Modified Monophosphate Prodrug of Remdesivir Parent Nucleoside (GS-441524). *Microbiology Spectrum* 2021; **9(3) (no pagination)**.
41. Wright PJ, Crameri G, Eaton BT. RNA synthesis during infection by Hendra virus: an examination by quantitative real-time PCR of RNA accumulation, the effect of ribavirin and the attenuation of transcription. *Archives of Virology* 2005; **150**(3): 521-32.
42. Porotto M, Orefice G, Yokoyama CC, et al. Simulating henipavirus multicycle replication in a screening assay leads to identification of a promising candidate for therapy. *Journal of Virology* 2009; **83(10)**: 5148-55.
43. Welch SR, Tilston NL, Lo MK, et al. Inhibition of Nipah virus by defective interfering particles. (Special Issue: Twenty years of Nipah virus research.). *Journal of Infectious Diseases* 2020; **221**(Suppl. 4): S460-S70.
44. Aljofan M, Lo MK, Rota PA, Michalski WP, Mungall BA. Off Label Antiviral Therapeutics for Henipaviruses: New Light Through Old Windows. *J Antivir Antiretrovir* 2010; **2**(1): 1-10.
45. Aljofan M, Sganga ML, Lo MK, et al. Antiviral activity of gliotoxin, gentian violet and brilliant green against Nipah and Hendra virus in vitro. *Virology Journal* 2009; **6 (no pagination)**.
46. Elshabrawy HA, Fan J, Haddad CS, et al. Identification of a Broad-Spectrum Antiviral Small Molecule against Severe Acute Respiratory Syndrome Coronavirus and Ebola, Hendra, and Nipah Viruses by Using a Novel High-Throughput Screening Assay. *Journal of Virology* 2014; **88**(8): 4353-65.
47. Hotard AL, He B, Nichol ST, Spiropoulou CF, Lo MK. 4′-Azidocytidine (R1479) inhibits henipaviruses and other paramyxoviruses with high potency. *Antiviral Research* 2017; **144**: 147-52.
48. Nguyen NM, Tran CN, Phung LK, et al. A randomized, double-blind placebo controlled trial of balapiravir, a polymerase inhibitor, in adult dengue patients. *J Infect Dis* 2013; **207**(9): 1442-50.
49. Janardhana V, Tachedjian M, Crameri G, Cowled C, Wang LF, Baker ML. Cloning, expression and antiviral activity of IFNgamma from the Australian fruit bat, Pteropus alecto. *Developmental and Comparative Immunology* 2012; **36(3)**: 610-8.
50. Liu SY, Aliyari R, Chikere K, et al. Interferon-inducible cholesterol-25-hydroxylase broadly inhibits viral entry by production of 25-hydroxycholesterol. *Immunity* 2013; **38**(1): 92-105.
51. Oey A, McClure M, Symons JA, et al. Lumicitabine, an orally administered nucleoside analog, in infants hospitalized with respiratory syncytial virus (RSV) infection: Safety, efficacy, and pharmacokinetic results. *PLoS One* 2023; **18**(7): e0288271.
52. Lo MK, Jordan PC, Stevens S, et al. Susceptibility of paramyxoviruses and filoviruses to inhibition by 2′-monofluoro- and 2′-difluoro-4′-azidocytidine analogs. *Antiviral Research* 2018; **153**: 101-13.
53. McCaskill JL, Marsh GA, Monaghan P, Wang LF, Doran T, McMillan NAJ. Potent Inhibition of Hendra Virus Infection via RNA Interference and Poly I: C Immune Activation. *PLoS ONE* 2013; **8(5) (no pagination)**.
54. Mohr EL, McMullan LK, Lo MK, et al. Inhibitors of cellular kinases with broad-spectrum antiviral activity for hemorrhagic fever viruses. *Antiviral Research* 2015; **120**: 40-7.
55. Mungall BA, Schopman NCT, Lambeth LS, Doran TJ. Inhibition of Henipavirus infection by RNA interference. *Antiviral Research* 2008; **80(3)**: 324-31.
56. Niedermeier S, Singethan K, Rohrer SG, et al. A Small-Molecule Inhibitor of Nipah Virus Envelope Protein-Mediated Membrane Fusion. *Journal of Medicinal Chemistry* 2009; **52**(14): 4257-65.
57. Pattabhi S, Wilkins CR, Dong R, et al. Targeting innate immunity for antiviral therapy through small molecule agonists of the RLR pathway. *Journal of Virology* 2016; **90(5)**: 2372-87.

58. Porotto M, Yi F, Moscona A, LaVan DA. Synthetic Protocells Interact with Viral Nanomachinery and Inactivate Pathogenic Human Virus. *PLoS ONE* 2011; **6**(3).
59. Pu J, He X, Xu W, et al. The Analogs of Furanyl Methylidene Rhodanine Exhibit Broad-Spectrum Inhibitory and Inactivating Activities against Enveloped Viruses, including SARS-CoV-2 and Its Variants. *Viruses* 2022; **14**(3).
60. Shrestha N, Gall FM, Mathieu C, et al. Highly Potent Host-Specific Small-Molecule Inhibitor of Paramyxovirus and Pneumovirus Replication with High Resistance Barrier. *Mbio* 2021; **12(6) (no pagination)**.
61. Tigabu B, Rasmussen L, White EL, et al. A BSL-4 high-throughput screen identifies sulfonamide inhibitors of Nipah virus. *Assay and Drug Development Technologies* 2014; **12(3)**: 155-61.
62. Wang YE, Park A, Lake M, et al. Ubiquitin-regulated nuclear-cytoplasmic trafficking of the Nipah virus matrix protein is important for viral budding. *PLoS Pathogens* 2010; **6(11) (no pagination)**.
63. Wolf MC, Freiberg AN, Zhang T, et al. A broad-spectrum antiviral targeting entry of enveloped viruses. *Proceedings of the National Academy of Sciences of the United States of America* 2010; **107(7)**: 3157-62.
